# Effectiveness of a Behavior Change Wheel–Informed WeChat-Based Messaging Intervention to Improve Medication Adherence After Percutaneous Coronary Intervention: 12-Week Quasi-Experimental Study

**DOI:** 10.2196/85192

**Published:** 2026-10-07

**Authors:** Yong Fang, Wenxiao Wu, Dili Chen, Zhili Jiang, Mingyue Zhang, Fanghong Dong, Xinyue Xiang, Lihua Huang

**Affiliations:** 1 Nursing Department, First Affiliated Hospital Zhejiang University, 79 Qingchun Road, Shangcheng District, Hangzhou City, 310053, China, 86 13867129329

**Keywords:** medication adherence, percutaneous coronary intervention, SMS text messaging, mobile health, behavior change techniques, capability, opportunity, and motivation–behavior model, COM-B model

## Abstract

**Background:**

Medication nonadherence after percutaneous coronary intervention (PCI) remains a major barrier to secondary prevention. Prior SMS text messaging interventions have shown inconsistent results, often limited to reminders without addressing behavioral or psychological determinants.

**Objective:**

This study aimed to evaluate the effectiveness of a theory-informed, WeChat-based messaging intervention for improving medication adherence and patient-reported outcomes after PCI.

**Methods:**

A nonrandomized quasi-experimental parallel-group study was conducted from July 2022 to March 2023 at a tertiary hospital in Hangzhou, China. Patients were allocated by ward admission to the intervention or control group. The intervention comprised 12-week WeChat-based medication reminders and theory-informed messages mapped to capability, opportunity, and motivation–behavior model domains and behavior change techniques. The primary outcome was medication adherence measured using the 8-item Morisky Medication Adherence Scale (MMAS-8); secondary outcomes were medication beliefs, self-efficacy, and disease-specific health status measured using the Beliefs About Medicines Questionnaire (BMQ)–Specific, Self-Efficacy for Appropriate Medication Use Scale, and Seattle Angina Questionnaire (SAQ), respectively. Outcomes were assessed at baseline and 12 weeks by blinded assessors and analyzed using baseline-adjusted analysis of covariance based on the observed outcome data for all 92 participants. Sensitivity analyses included a per-protocol analysis restricted to the 87 participants who completed the full assigned care protocol and a difference-in-differences analysis comparing changes from baseline to 12 weeks between groups.

**Results:**

Of 180 patients screened, 92 (51.1%) were enrolled, of whom all completed the 12-week outcome assessment and 87 (94.6%) completed the full assigned care protocol. At 12 weeks, medication adherence was higher in the intervention group than in the control group (adjusted mean MMAS-8 score 7.40, SE 0.05 vs 6.22, SE 0.10; adjusted mean difference 1.18, 95% CI 0.96‐1.40; *P*<.001). Secondary outcomes generally favored the intervention, including the BMQ necessity (adjusted mean difference 1.62, 95% CI 1.06‐2.17) and concerns (adjusted mean difference −3.25, 95% CI −3.87 to −2.63) subscales, medication self-efficacy (adjusted mean difference 4.04, 95% CI 3.21‐4.87), and the SAQ summary score (adjusted mean difference 6.13, 95% CI 4.72‐7.53; *P*<.001 in all cases). SAQ treatment satisfaction did not differ significantly between groups (adjusted mean difference 0.40, 95% CI −2.02 to 2.82; *P*=.74). Both the per-protocol and difference-in-differences sensitivity analyses yielded findings consistent with the primary analysis, supporting the robustness of the results.

**Conclusions:**

A theory-informed, WeChat-based messaging intervention was associated with improvements in medication adherence, medication beliefs, self-efficacy, and disease-specific health status after PCI. Larger, adequately powered randomized trials with longer follow-up are needed to confirm these findings.

## Introduction

Percutaneous coronary intervention (PCI) is the standard revascularization strategy for eligible patients with coronary heart disease (CHD), offering rapid relief of ischemic symptoms by reopening stenotic arteries [1]. However, PCI does not halt the progression of atherosclerosis. Patients remain at risk of restenosis and recurrent cardiovascular events, with restenosis rates of approximately 10% [2,3]. Accordingly, contemporary clinical guidelines recommend long-term, guideline-directed pharmacotherapy for secondary prevention after PCI to reduce recurrent ischemic events and improve long-term clinical outcomes [1,4].

Medication adherence refers to the extent to which patients take their medications as prescribed. In practice, adherence after PCI remains suboptimal. In the China PEACE (Patient-Centered Evaluative Assessment of Cardiac Events) cohort [5], nearly 30% of patients did not achieve good medication adherence during the first month after discharge, and only approximately 50% remained adherent to aspirin and P2Y12 inhibitors at 1 year. Similar patterns have been reported internationally. Registry studies from the United States and Europe indicate that only 50% to 70% of patients remain adherent to dual antiplatelet therapy at 1 year, with many discontinuing treatment prematurely [6-9]. Adherence is often even lower in low- and middle-income countries because of financial constraints, structural barriers, and limited health literacy [10,11]. Nonadherence is associated with an increased risk of adverse cardiovascular outcomes [12-14], underscoring the need for scalable, accessible, and potentially cost-effective strategies to support long-term medication adherence across diverse settings [15].

Mobile phone–based interventions, particularly SMS text messaging, offer promise because they are inexpensive, widely accessible, and acceptable to patients [16,17]. However, evidence from prior SMS text messaging interventions after PCI is heterogeneous. Some studies have documented improved adherence [18-20], whereas others have reported no benefit or only short-term improvements [21-23]. Many existing interventions rely on generic reminders or educational content without adequately addressing the specific capability, opportunity, and motivational barriers underlying medication nonadherence. Message timing and content may also be insufficiently aligned with patients’ medication routines and practical needs [17]. More fundamentally, the behavioral rationale for individual messages is often unclear. It is not always evident which adherence barrier a message is intended to address, which behavior change mechanism it targets, or how that mechanism is reflected in the message content and delivery [24,25]. Consequently, an important knowledge gap concerns how identified adherence barriers can be translated into theoretically informed, specific, and reproducible messaging components.

The capability, opportunity, and motivation–behavior (COM-B) model provides a framework for identifying the determinants of medication-taking behavior. Capability refers to the knowledge and skills required to take medications appropriately; opportunity encompasses environmental resources, cues, and social support; and motivation includes beliefs, intentions, and automatic processes that influence behavior. The Behavior Change Wheel (BCW) builds on this behavioral diagnosis by linking COM-B determinants to intervention functions and behavior change techniques (BCTs), which are the observable and reproducible components through which an intervention is delivered [26]. In our prior work, we applied the COM-B model to systematically identify barriers to and facilitators of medication adherence among patients following PCI [27]. Capability-related barriers included limited health literacy and uncertainty about side effects or medication adjustments. Opportunity-related barriers included difficulties with medication refills, competing life demands, and limited communication with clinicians. Motivation-related barriers included declining perceptions of cardiovascular risk and concerns about bleeding or polypharmacy [27]. Although the BCW has been applied in lifestyle and chronic disease interventions [28-30], its use in cardiovascular pharmacotherapy after PCI remains limited. Few studies have documented a transparent pathway from COM-B–identified adherence barriers to selected BCTs and specific messaging components in post-PCI care.

To address this gap, we developed MedText-PCI, a BCW-informed WeChat-based messaging intervention targeting capability, opportunity, and motivation. This study extends previous work by showing how adherence barriers identified through the COM-B were linked to BCW intervention functions and BCTs and then translated into specific message content and delivery strategies. It also evaluated medication adherence together with relevant behavioral and health outcomes. We conducted a 12-week quasi-experimental study to address two research questions: compared with usual care, was MedText-PCI associated with (1) higher medication adherence at 12 weeks and (2) more favorable medication beliefs, medication self-efficacy, and disease-specific health status? Disease-specific health status encompassed angina symptoms, physical limitations, treatment satisfaction, and quality of life.

## Methods

### Design

This was a 12-week, nonrandomized quasi-experimental parallel-group trial.

### Participants

Patients were recruited from the cardiology wards of a tertiary general hospital in Hangzhou, Zhejiang Province, China, between July 2022 and March 2023. Potentially eligible participants were identified through daily review of ward admission records. Using consecutive sampling, trained research staff screened all patients admitted to the cardiology wards who had undergone PCI during the recruitment period against the prespecified eligibility criteria. Eligible patients received written information about the study and provided written informed consent before enrollment. Eligible participants met all the following inclusion criteria: (1) age of 18 to 75 years, (2) diagnosis of CHD according to World Health Organization criteria and status after PCI, (3) patient and family willingness to participate and cooperate, and (4) ownership of a mobile phone with the ability to use WeChat and telephone functions. Participants meeting any of the following exclusion criteria were not enrolled: (1) advanced heart failure (New York Heart Association class IV); (2) severe comorbid disease involving major organs (eg, brain, liver, or kidney) or other serious somatic conditions; (3) illiteracy or visual or hearing impairments precluding reading SMS text messages; and (4) cognitive impairment, communication disorders, or major psychiatric illness. Participation was voluntary and uncompensated.

### Sample Size

On the basis of the pilot 8-item Morisky Medication Adherence Scale (MMAS-8) data (Multimedia Appendix 1), an expected between-group mean difference of 0.78 points at the 1-month follow-up and a pooled SD of 1.01 were assumed. Using PASS (version 15.0; NCSS, LLC), the sample size was calculated for a 2-tailed independent-sample *t* test with an α level of .05, power of 80%, and a 1:1 allocation ratio. The calculation indicated that 56 participants were required to complete the study, with 28 participants per group. After allowing for an anticipated 10% loss to follow-up, the recruitment target was set at 64 participants, with 32 participants per group. Ultimately, 92 participants were enrolled, including 46 (50%) participants in each group, thereby exceeding the prespecified recruitment target.

### Group Assignment and Blinding

Participants were allocated to the intervention or control condition according to the cardiology ward to which they were admitted, with one ward assigned to each study condition. Ward-based allocation was used to maintain separation between study conditions, minimize intervention contamination, and preserve routine clinical workflow. Individual randomization within the same ward was considered less appropriate because participants shared the same clinical environment and ward-based care team, potentially increasing contamination across study conditions. Cluster randomization was not feasible because only 2 eligible wards were available, resulting in a single cluster per condition. Given the behavioral nature of the intervention, participants and treating clinicians could not be masked. Outcome assessors and data collection personnel were masked to allocation; coded participant identifiers were used, intervention records were inaccessible to assessors, and intervention delivery and outcome assessment were conducted by separate study personnel to minimize assessment bias.

### Intervention

Participants in the control group received standard postoperative counseling on medication, physical activity, and diet together with a printed education booklet on post-PCI care (Multimedia Appendix 2).

In addition to usual care, participants in the intervention group received a BCW-informed WeChat-based messaging intervention initiated immediately after hospital discharge. The program had 2 components. First, medication reminders were sent approximately 30 minutes before each participant’s usual dosing time based on baseline self-reported regimens [31]. Second, 1 nonreminder message was delivered daily at 8 AM for 12 weeks (Figure 1).

**Figure 1.**
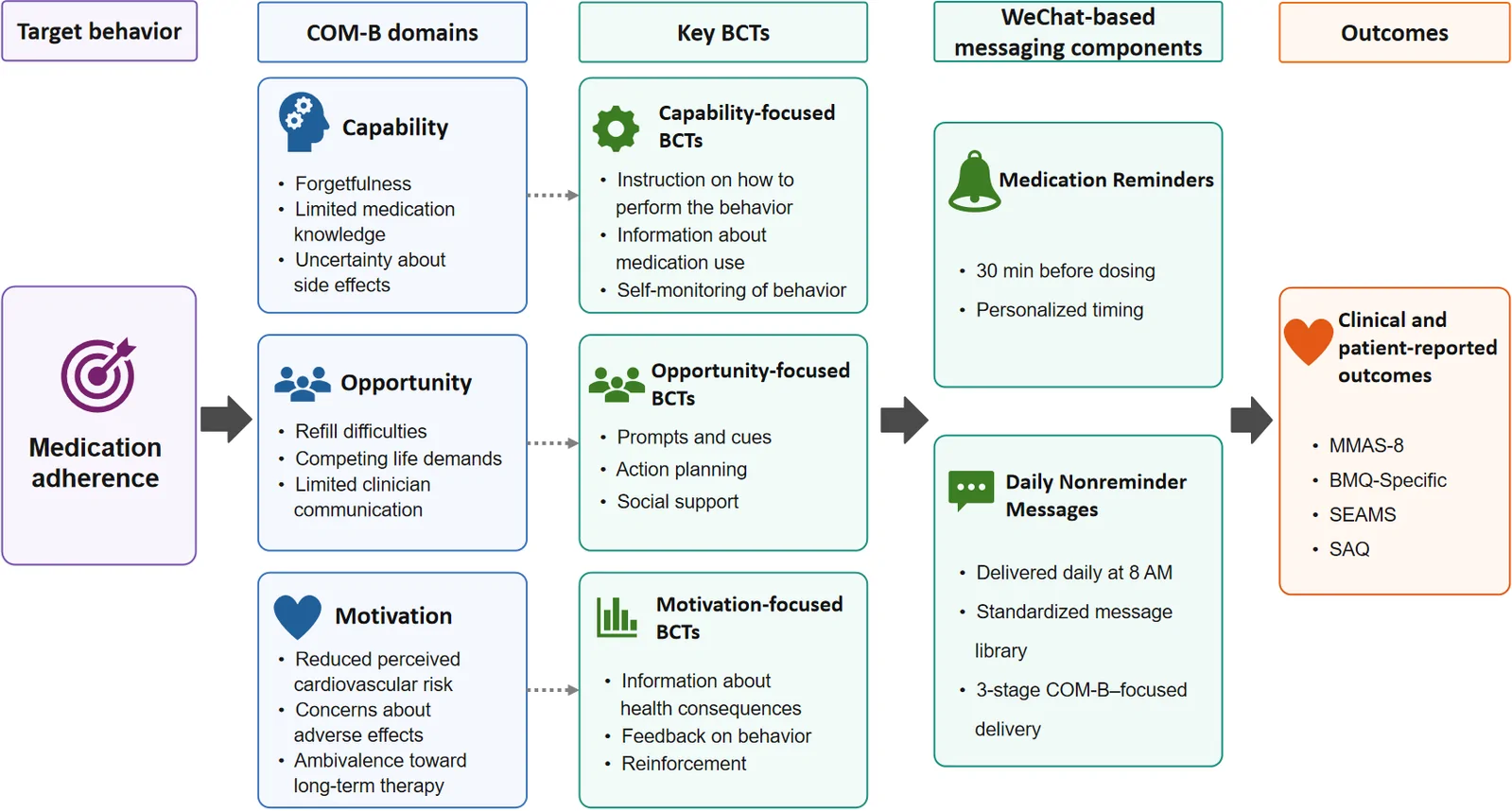
Mechanistic framework of a Behavior Change Wheel–informed WeChat messaging intervention after percutaneous coronary intervention. BCT: behavior change technique; BMQ: Beliefs About Medicines Questionnaire; COM-B: capability, opportunity, and motivation–behavior; MMAS-8: 8-item Morisky Medication Adherence Scale; SAQ: Seattle Angina Questionnaire; SEAMS: Self-Efficacy for Appropriate Medication Use Scale.

Nonreminder messages were drawn from a standardized message library (Multimedia Appendix 3) [26,27]. Development was guided by the BCW. Adherence barriers were first identified using the COM-B framework. Each barrier was then mapped to appropriate intervention functions and associated BCTs. Candidate messages were drafted and refined by an expert panel according to these mappings. Wording and usability were informed by patient feedback from pilot-testing. The final library covered medication information, management of adverse effects, benefits of adherence, self-monitoring strategies, behavioral techniques, and prompts for social support. Detailed COM-B–intervention function–BCT mapping and representative message examples are provided in Multimedia Appendix 3 [26,27]. The complete message library [32], which was previously published as part of our intervention development work, is also included for transparency and reproducibility.

The 12-week message delivery schedule, including reminder timing and the stage-specific distribution of nonreminder messages across capability-, motivation-, and opportunity-focused phases, is detailed in Multimedia Appendix 4. Weeks 1 to 3 focused on capability building, including PCI and medication knowledge, side effect management, and self-monitoring skills. Weeks 4 to 6 emphasized motivation by reinforcing adherence benefits, providing behavioral feedback, and strengthening psychological and social support. Weeks 7 to 12 targeted opportunity through habit formation, environmental cues, planning for travel or busy schedules, and self-reward to support long-term adherence. Participants could pause or opt out at any time and were instructed to call a 24-hour hotline for adverse events or worsening symptoms.

### Measures

#### Outcomes

All outcomes were evaluated at baseline and at 12 weeks by trained assessors who were independent of the intervention team and blinded to group allocation. At 12 weeks after discharge, data were collected either via telephone or during face-to-face visits according to participant availability.

##### Primary Outcome

The primary outcome was medication adherence, defined as the extent to which participants took their medications as prescribed. It was assessed using the MMAS-8 [33-36]. The first 7 items use dichotomous “yes” or “no” responses and are scored according to the standard MMAS-8 coding algorithm. The eighth item uses a 5-point response scale ranging from “never” to “all the time,” scored as 1.00, 0.75, 0.50, 0.25, and 0, respectively. Total scores range from 0 to 8, with scores below 6 indicating low adherence, scores from 6 to less than 8 indicating medium adherence, and a score of 8 indicating high adherence. The MMAS-8 has demonstrated acceptable internal consistency, with a reported Cronbach α coefficient of 0.83.

##### Secondary Outcomes

###### Medication Beliefs

Medication beliefs referred to participants’ perceived need for their prescribed medications and their concerns about potential adverse consequences. They were assessed using the Chinese version of the Beliefs About Medicines Questionnaire (BMQ)–Specific. The questionnaire contains 10 items divided equally between the necessity and concerns subscales. Each item is rated on a 5-point Likert scale, producing a score of 5 to 25 for each subscale. The necessity-concerns differential was calculated by subtracting the concerns score from the necessity score, with a higher differential indicating that perceived necessity outweighed medication-related concerns to a greater extent. The Chinese version has demonstrated acceptable internal consistency, with a reported Cronbach α coefficient of 0.77 [37].

###### Medication Self-Efficacy

Medication self-efficacy referred to participants’ confidence in taking medications as prescribed under difficult or uncertain circumstances. It was assessed using the Chinese version of the 13-item Self-Efficacy for Appropriate Medication Use Scale (SEAMS) [38]. Items are rated on a 3-point scale (1=“not confident”; 2=“somewhat confident”; 3=“very confident”). Total scores range from 13 to 39, with higher scores indicating greater medication self-efficacy. The Chinese version has demonstrated a Cronbach α coefficient of 0.934, a test-retest reliability coefficient of 0.932, and a content validity index of 0.913.

### Disease-Specific Health Status

Disease-specific health status was assessed using the 19-item Seattle Angina Questionnaire (SAQ). The SAQ comprises 5 domains: physical limitation, angina stability, angina frequency, treatment satisfaction, and disease-related quality of life. Scores for each domain were transformed to a scale from 0 to 100 using the following formula: (observed score – lowest possible score)/(highest possible score – lowest possible score) × 100. Higher scores indicate better disease-specific health status, including fewer angina-related limitations and better functioning and quality of life. The SAQ has demonstrated acceptable internal consistency, with a reported Cronbach α coefficient of 0.76 [39].

### Statistical Analysis

All 92 participants were analyzed according to their initial group allocation using observed data; no imputation was required because outcome data were complete. For continuous outcomes, between-group differences at 12 weeks were estimated using analysis of covariance (ANCOVA), with treatment group as the fixed factor and the baseline value of the corresponding outcome as a covariate. Homogeneity of regression slopes was assessed using the treatment group–by-baseline interaction, and linearity was assessed by adding a quadratic baseline term. Residual normality and homoscedasticity were assessed using the Jarque-Bera and Breusch-Pagan tests, respectively. Because heteroscedasticity was identified in several models, HC3 heteroscedasticity-robust SEs were used for all final models. When homogeneity of regression slopes was not supported, the interaction term was retained, and adjusted group estimates were calculated at the overall mean baseline value. Detailed ANCOVA diagnostic results are presented in Multimedia Appendix 5. Sensitivity analyses included a per-protocol analysis restricted to the 87 participants who completed the full assigned care protocol and a difference-in-differences analysis comparing individual changes from baseline to 12 weeks between groups. The per-protocol analysis used the same ANCOVA models as the primary analysis, whereas the difference-in-differences analysis used HC3 robust SEs. All statistical tests were 2 sided, with a *P* value below .05 indicating statistical significance. Statistical analyses were conducted using SPSS Statistics (version 25; IBM Corp) and Python (version 3.12; Python Software Foundation).

### Ethical Considerations

This study received ethics approval from the ethics committee of the First Affiliated Hospital of Zhejiang University (11T20220185B). Written informed consent was obtained from all participants. All procedures complied with good clinical practice and the Declaration of Helsinki. Only deidentified data were analyzed, and access was restricted to the study team. Data handling complied with national data protection regulations and institutional data security policies.

## Results

### Characteristics of the Study Population

Between July 2022 and March 2023, a total of 180 consecutive patients were screened for eligibility. Of these 180 patients, 88 (48.9%) were not enrolled: 56 (63.6%) did not meet the eligibility criteria, 23 (26.1%) declined to participate, and 9 (10.2%) were discharged or transferred before enrollment or did not complete the baseline assessment. The remaining 92 participants were nonrandomly allocated by ward admission to the intervention group (n=46, 50%) or the control group (n=46, 50%; Figure 2). Assigned care was completed by 95.7% (44/46) of the participants in the intervention group and 93.5% (43/46) of the participants in the control group. Overall, 94.6% (87/92) of the participants completed the full assigned care protocol. Although 5.4% (5/92) of the participants did not complete the full protocol, all completed the 12-week outcome assessment. Therefore, all 92 participants were included in the outcome analyses. Baseline characteristics were similar between groups (Table 1). The mean age was 61.11 (SD 8.52) years in the intervention group and 61.74 (SD 5.60) years in the control group. Educational attainment was generally low, with 58.7% (54/92) having junior high education or lower and 15.2% (14/92) having a college education or higher. Most participants were retired (57/92, 62%) or employed (35/92, 38%). A total of 16.3% (15/92) had a monthly per capita household income of less than ¥3000 (¥1=US $0.15 as of August 18, 2026), 52.2% (48/92) had an income of ¥3000 to ¥5000, and 31.5% (29/92) had an income above ¥5000. Comorbid chronic disease was present in 59.8% (55/92) of participants, and 53.3% (49/92) were newly diagnosed with CHD.

**Figure 2.**
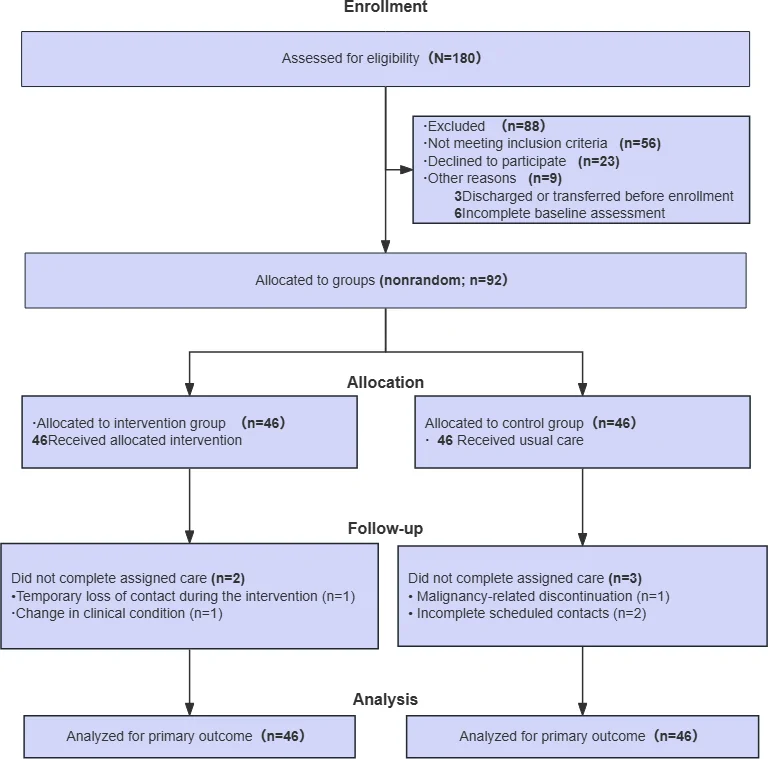
Study flow diagram. All participants completed the 12-week outcome assessment and were included in the primary analysis.

**Table 1.** Baseline characteristics of the study participants (N=92).

Characteristics	Total	Control (n=46)	Intervention (n=46)	*P* value
Sex, n (%)				.20^a^
Male	73 (79.3)	34 (73.9)	39 (84.8)	
Female	19 (20.7)	12 (26.1)	7 (15.2)	
Age (y), mean (SD)	61.42 (7.18)	61.74 (5.60)	61.11 (8.52)	.68^b^
Educational level, n (%)				.35^a^
Junior high school or lower	54 (58.7)	29 (63)	25 (54.3)	
High school	24 (26.1)	9 (19.6)	15 (32.6)	
College or higher	14 (15.2)	8 (17.4)	6 (13)	
Occupation, n (%)				.83^a^
Employed	35 (38)	17 (37)	18 (39.1)	
Retired	57 (62)	29 (63)	28 (60.9)	
Monthly household income per capita (¥; ¥1=US $0.15 as of August 18, 2026), n (%)				.91^a^
<3000	15 (16.3)	7 (15.2)	8 (17.4)	
3000‐5000	48 (52.2)	25 (54.3)	23 (50)	
>5000	29 (31.5)	14 (30.4)	15 (32.6)	
Comorbid chronic disease, n (%)				.83^a^
Yes	55 (59.8)	28 (60.9)	27 (58.7)	
No	37 (40.2)	18 (39.1)	19 (41.3)	
First diagnosis of coronary heart disease, n (%)				.30^a^
Yes	49 (53.3)	27 (58.7)	22 (47.8)	
No	43 (46.7)	19 (41.3)	24 (52.2)	

a Pearson chi-square test.

b Welch *t* test.

### Medication Adherence and Secondary Outcomes

ANCOVA diagnostic analyses supported the linearity assumption for all outcome models. Homogeneity of regression slopes was supported for all outcomes except MMAS-8 and SAQ angina frequency, for which the group-by-baseline interactions were significant (*P*<.001 and *P*=.03, respectively). Departures from residual normality or homoscedasticity were identified in several models; therefore, HC3 heteroscedasticity-robust SEs were used throughout. Detailed diagnostic results can be found in Multimedia Appendix 5.

At the overall mean baseline MMAS-8 score of 6.05, the adjusted 12-week mean MMAS-8 score was higher in the intervention group than in the control group (7.40, SE 0.05 vs 6.22, SE 0.10; adjusted mean difference 1.18, 95% CI 0.96-1.40; *P*<.001; Table 2).

**Table 2.** Primary and secondary outcomes at 12 weeks by group (analysis of covariance adjusted for baseline of each measure)^a^.

Outcomes	Intervention group (n=46), adjusted mean (SE; 95% CI)	Control group (n=46), adjusted mean (SE; 95% CI)	Adjusted mean difference (95% CI)	*P* value
Primary outcome				
MMAS-8^b^ score (0-8)	7.40 (0.05; 7.31 to 7.49)	6.22 (0.10; 6.02 to 6.42)	1.18 (0.96 to 1.40)	<.001
Secondary outcomes				
BMQ^c^ score				
Necessity (5-25)	22.46 (0.18; 22.11 to 22.81)	20.84 (0.22; 20.41 to 21.28)	1.62 (1.06 to 2.17)	<.001
Concerns (5-25)	11.99 (0.25; 11.51 to 12.48)	15.25 (0.20; 14.86 to 15.63)	−3.25 (−3.87 to −2.63)	<.001
Necessity – concerns differential	10.54 (0.37; 9.80 to 11.27)	5.53 (0.32; 4.89 to 6.17)	5.01 (4.03 to 5.98)	<.001
SEAMS^d^ score (13-39)	28.10 (0.39; 27.32 to 28.87)	24.06 (0.15; 23.75 to 24.36)	4.04 (3.21 to 4.87)	<.001
SAQ^e^ score				
Physical limitation (0-100)	86.01 (1.05; 83.93 to 88.10)	77.80 (0.47; 76.86 to 78.75)	8.21 (5.90 to 10.53)	<.001
Angina stability (0-100)	73.34 (1.93; 69.50 to 77.17)	60.36 (2.07; 56.26 to 64.47)	12.98 (7.35 to 18.60)	<.001
Angina frequency (0-100)	93.38 (0.70; 91.99 to 94.77)	85.55 (1.06; 83.44 to 87.66)	7.83 (5.30 to 10.36)	<.001
Treatment satisfaction (0-100)	83.83 (0.81; 82.22 to 85.44)	83.43 (0.90; 81.65 to 85.21)	0.40 (−2.02 to 2.82)	.74
Quality of life (0-100)	71.60 (1.34; 68.93 to 74.27)	64.27 (1.21; 61.86 to 66.68)	7.33 (3.70 to 10.96)	<.001
Summary score (0-100)	75.30 (0.63; 74.04 to 76.56)	69.17 (0.30; 68.58 to 69.77)	6.13 (4.72 to 7.53)	<.001

a Values are adjusted means estimated at the overall mean baseline score. Group-by-baseline interactions were retained for the 8-item Morisky Medication Adherence Scale and Seattle Angina Questionnaire angina frequency. Differences were calculated as intervention minus control. HC3 robust SEs were used.

b MMAS-8: 8-item Morisky Medication Adherence Scale.

c BMQ: Beliefs About Medicines Questionnaire.

d SEAMS: Self-Efficacy for Appropriate Medication Use Scale.

e SAQ: Seattle Angina Questionnaire.

Secondary outcome findings were generally consistent with those for the primary outcome. Compared with the control group, the intervention group had higher BMQ necessity subscale scores (adjusted mean difference 1.62, 95% CI 1.06-2.17), lower BMQ concerns subscale scores (adjusted mean difference −3.25, 95% CI −3.87 to −2.63), and a higher necessity-concerns differential (adjusted mean difference 5.01, 95% CI 4.03-5.98; *P*<.001 in all cases). SEAMS scores were also higher in the intervention group (adjusted mean difference 4.04, 95% CI 3.21-4.87; *P*<.001).

For the SAQ, the intervention group had more favorable adjusted scores for physical limitation, angina stability, angina frequency, and quality of life. Treatment satisfaction did not differ significantly between groups (adjusted mean difference 0.40, 95% CI −2.02 to 2.82; *P*=.74). The SAQ summary score was higher in the intervention group (adjusted mean difference 6.13, 95% CI 4.72-7.53; *P*<.001).

### Sensitivity Analyses

Sensitivity analyses supported the robustness of the primary findings. In the per-protocol analysis of the 87 participants who completed the full assigned care protocol, the adjusted between-group difference in MMAS-8 score was 1.14 (95% CI 0.92‐1.36; *P*<.001). The difference-in-differences analysis also favored the intervention (between-group difference in change=1.08, 95% CI 0.75‐1.40; *P*<.001). Findings for the secondary outcomes were generally consistent with those of the primary analysis, whereas SAQ treatment satisfaction did not differ significantly between groups (95% CI −1.93 to 2.82; *P*=.71). Detailed results can be found in Multimedia Appendix 6.

## Discussion

### Principal Findings

In this quasi-experimental study, a WeChat-based messaging intervention was associated with higher medication adherence and more favorable medication beliefs, self-efficacy, and disease-specific health status among patients after PCI. MedText-PCI was designed to address both unintentional and intentional barriers to medication taking, and the observed differences extended beyond adherence to broader behavioral and health outcomes. Unlike generic or reminder-dominant approaches, MedText-PCI explicitly mapped adherence barriers to BCTs and corresponding message components targeting capability, opportunity, and motivation. The assessment of medication beliefs and self-efficacy alongside adherence provided preliminary insights into outcomes aligned with these behavioral targets.

First, the observed improvement in medication adherence may reflect the intervention’s simultaneous targeting of unintentional and intentional barriers to medication taking. Contemporary reviews indicate that medication nonadherence reflects both unintentional execution failures, such as forgetting and routine disruption, and intentional decisions shaped by medication beliefs and motivation [40]. MedText-PCI was explicitly designed to address both pathways. Timely prompts before dosing served as contextual cues intended to reduce reliance on memory and help integrate medication taking into daily routines, thereby supporting opportunity within the COM-B model [41]. On the intentional pathway, nonadherence may reflect doubts about medication necessity, concerns about adverse effects, and ambivalence toward long-term therapy. Educational messages were designed to improve understanding of PCI medications and the practical management of side effects, whereas motivational content sought to reinforce perceived necessity and address medication-related concerns. The observed widening of the necessity-concerns differential was consistent with changes in medication-related motivation [42]. Improvements in self-efficacy may additionally reflect greater psychological capability for managing medication taking [43].

Comparison with previous SMS text messaging–based cardiovascular interventions provides important context for interpreting our findings. Findings from earlier cardiovascular trials have been mixed. The original Text4Heart trial [19] reported an improvement in self-reported medication adherence, whereas TXT2HEART [21], TEXTMEDS (Text Messages to Improve Medication Adherence and Secondary Prevention) [22], Text4Heart II [23], and the StAR (SMS-Text Adherence Support) trial [44] found no clear improvement in adherence relative to usual care. Against this background, the higher MMAS-8 scores observed with MedText-PCI provide preliminary evidence that a messaging intervention focused specifically on medication taking after PCI may support short-term adherence. Several differences may help contextualize these findings. Unlike interventions that rely mainly on medication reminders or general supportive messages, MedText-PCI combined medication-timed reminders with educational and motivational content. These messages were developed by linking COM-B–identified adherence barriers to BCW intervention functions, BCTs, and specific message components [32]. By comparison, TXT2HEART [21] used a longer intervention with progressively reduced message frequency, TEXTMEDS [22] delivered broader weekly supportive messages after acute coronary syndrome, and Text4Heart II [23] evaluated adherence primarily using pharmacy-based medication possession ratios. These differences in intervention intensity, behavioral targets, adherence measurement, follow-up duration, and usual care conditions may partly explain variation across studies. However, they do not establish that the MedText-PCI design is more effective, particularly because the present study was nonrandomized, relied on self-reported adherence, and assessed outcomes after only 12 weeks.

Second, the favorable pattern observed with MedText-PCI extended beyond medication adherence to theoretically relevant psychological and health-related outcomes. Previous messaging studies have examined similar outcomes, although their findings have been mixed. Text4Heart found no clear between-group differences in self-efficacy or illness perceptions despite improving self-reported adherence [19], whereas Park et al [45] reported no significant improvement in medication self-efficacy following reminder and educational SMS text messages among patients with CHD. Similarly, Txt2Prevent found no significant effects on cardiac self-efficacy or generic health-related quality of life after acute coronary syndrome [46], and neither the StAR trial [44] nor Text4Heart II [23] demonstrated clear benefits for generic health status or quality of life outcomes. TEXT ME (Tobacco, Exercise, and Diet Messages) improved several cardiovascular risk factors and lifestyle behaviors but did not assess medication-specific beliefs or self-efficacy [47]. In MedText-PCI, medication beliefs and self-efficacy were assessed as outcomes proximal to the intervention’s proposed behavioral targets, whereas disease-specific health status represented a potential downstream health outcome. The more favorable scores observed for these outcomes are consistent with the intervention’s theoretical rationale. However, treatment satisfaction did not differ between groups, suggesting that the intervention may have had limited influence on the relational and service attributes captured by this SAQ domain, such as interpersonal care, continuity, and access. High baseline scores and the relatively short 12-week intervention period may have further limited the potential for improvement. These findings suggest that digital adherence support may complement rather than replace clinician-led relational care [48]. Future versions could therefore incorporate bidirectional communication with the care team and assess patient experience outcomes that are more sensitive to communication and partnership [49,50]. Moreover, because these variables were assessed concurrently and were largely self-reported, the findings do not establish that changes in beliefs or self-efficacy mediated the observed adherence difference.

The clinical relevance of the observed between-group differences, however, requires separate consideration. No validated minimal clinically important difference (MCID) for the MMAS-8 has been established specifically for patients with CHD. Although the adjusted between-group difference was 1.18 points, both adjusted means remained within the conventional medium-adherence range [51]. Thus, the finding indicates improved self-reported adherence, but its clinical importance cannot be established based on an MMAS-8 MCID [52]. For the SAQ, the mean differences in physical limitation (8.21 points) and the summary score (6.13 points) met commonly cited clinically important thresholds of approximately 8 and 5 points, respectively [53,54]. The other SAQ domain differences did not reach their corresponding thresholds. However, because these thresholds primarily describe within-person changes, caution is warranted when applying them to between-group differences. Better medication adherence may plausibly improve long-term cardiovascular outcomes. Nevertheless, because this 12-week pilot study did not assess cardiovascular events, the observed improvement in MMAS-8 scores cannot be translated into a specific cardiovascular risk reduction. Larger, longer-term randomized trials using objective adherence measures and adjudicated cardiovascular outcomes are therefore needed.

### Implications and Future Directions

Delivery through WeChat may have facilitated access because the platform was familiar to participants, but this context also limits generalizability. Although the behavioral content could be adapted to SMS text messaging, WhatsApp, patient portals, or other platforms, its effectiveness may vary according to platform functions, costs, accessibility, cultural expectations, and integration with health care systems [55]. Digital exclusion also warrants attention, particularly among older adults and people with limited digital literacy, restricted internet access, or shared devices [56]. Future studies should therefore evaluate the intervention across platforms and settings while considering simplified interfaces, user training, caregiver support, and alternative delivery options.

Commercial messaging platforms also raise privacy and data security concerns because notifications displayed on locked screens or shared devices may disclose medication use or health conditions [57]. Implementation should minimize sensitive content; provide users with control over notification previews and opting out; and establish appropriate procedures for consent, access, data retention, and deletion. Platform-specific governance, regulatory compliance, and users’ privacy preferences should also be evaluated [58]. Given the 12-week follow-up, larger and longer-term trials should examine the durability of intervention effects, equity, privacy, cost-effectiveness, and integration with clinical workflows and caregiver support.

### Limitations

This study has several limitations. First, despite enrollment exceeding the prespecified minimum, the nonrandomized design, modest sample size, and single-center setting limit causal inference and generalizability. Ward-based allocation and the inability to mask participants and clinicians may have introduced ward-level confounding, selection and performance biases, Hawthorne effects, and differential attention despite the use of common institutional care procedures and separate outcome assessment. Second, the 12-week intervention and follow-up periods precluded evaluation of the durability of the observed differences or their association with longer-term clinical outcomes. Third, medication adherence was self-reported and may have been affected by recall and social desirability biases; the absence of objective adherence measures also prevented corroboration of the MMAS-8 findings. The psychological and health status outcomes were assessed concurrently and were largely self-reported. These assessments cannot determine whether changes in these outcomes mediated the observed adherence difference. Finally, the uniform message library and delivery schedule provided limited scope for personalization, and privacy perceptions and data security outcomes were not formally assessed. Generalizability may also be limited among individuals with lower digital literacy or restricted digital access and in settings where WeChat is not routinely used.

### Conclusions

In this quasi-experimental study, a WeChat-based messaging intervention was associated with improvements in medication adherence, medication beliefs, self-efficacy, and disease-specific health status among post-PCI patients. By addressing cognitive, motivational, and social support–related barriers in addition to forgetfulness, the program extended beyond reminder-only approaches and may offer a low-intensity strategy for supporting secondary prevention. Larger, adequately powered multicenter randomized trials with longer follow-up are needed to evaluate the intervention’s effectiveness, sustainability, cost-effectiveness, and implementation across diverse settings using objective adherence measures. Future research could examine whether AI can identify patient-specific barriers to medication adherence and use this information to select and deliver the most appropriate messages from an established message library.

## Supplementary material

10.2196/85192 Multimedia Appendix 1 Pilot data on score changes from discharge to 1-month follow-up among participants in the intervention and control groups..docx

10.2196/85192 Multimedia Appendix 2 Patient education booklet on medication management and secondary prevention after percutaneous coronary intervention.

10.2196/85192 Multimedia Appendix 3 Development process of SMS messages based on the Behavior Change Wheel framework for the MedText–percutaneous coronary intervention.

10.2196/85192 Multimedia Appendix 4 WeChat text-messaging delivery plan for the MedText–percutaneous coronary intervention

10.2196/85192 Multimedia Appendix 5 Diagnostic assessment of ANCOVA assumptions using observed data..docx

10.2196/85192 Multimedia Appendix 6 Detailed results of sensitivity analyses, including per-protocol and difference-in-differences analyses.

## Abbreviations

ANCOVA: analysis of covariance
BCT: behavior change technique
BCW: Behavior Change Wheel
BMQ: Beliefs About Medicines Questionnaire
CHD: coronary heart disease
COM-B: capability, opportunity, and motivation–behavior
MCID: minimal clinically important difference
MMAS-8: 8-item Morisky Medication Adherence Scale
PCI: percutaneous coronary intervention
PEACE: Patient-Centered Evaluative Assessment of Cardiac Events
SAQ: Seattle Angina Questionnaire
SEAMS: Self-Efficacy for Appropriate Medication Use Scale
StAR: SMS-Text Adherence Support
TEXT ME: Tobacco, Exercise, and Diet Messages
TEXTMEDS: Text Messages to Improve Medication Adherence and Secondary Prevention
