# Supplementary material for: Effectiveness of a Behavior Change Wheel–Informed WeChat-Based Messaging Intervention to Improve Medication Adherence After Percutaneous Coronary Intervention: 12-Week Quasi-Experimental Study
Source: J Med Internet Res. 2026 Oct 7;28:e85192. doi: 10.2196/85192 (PMC13645199; doi:10.2196/85192)
Supplement: Supplementary file 1 [file jmir-v28-e85192-s002.pdf]

I25.103①：冠状动脉粥样硬化性心脏病

I25.103 ①：CORONARY ATHEROSCLEROTIC HEART DISEASE

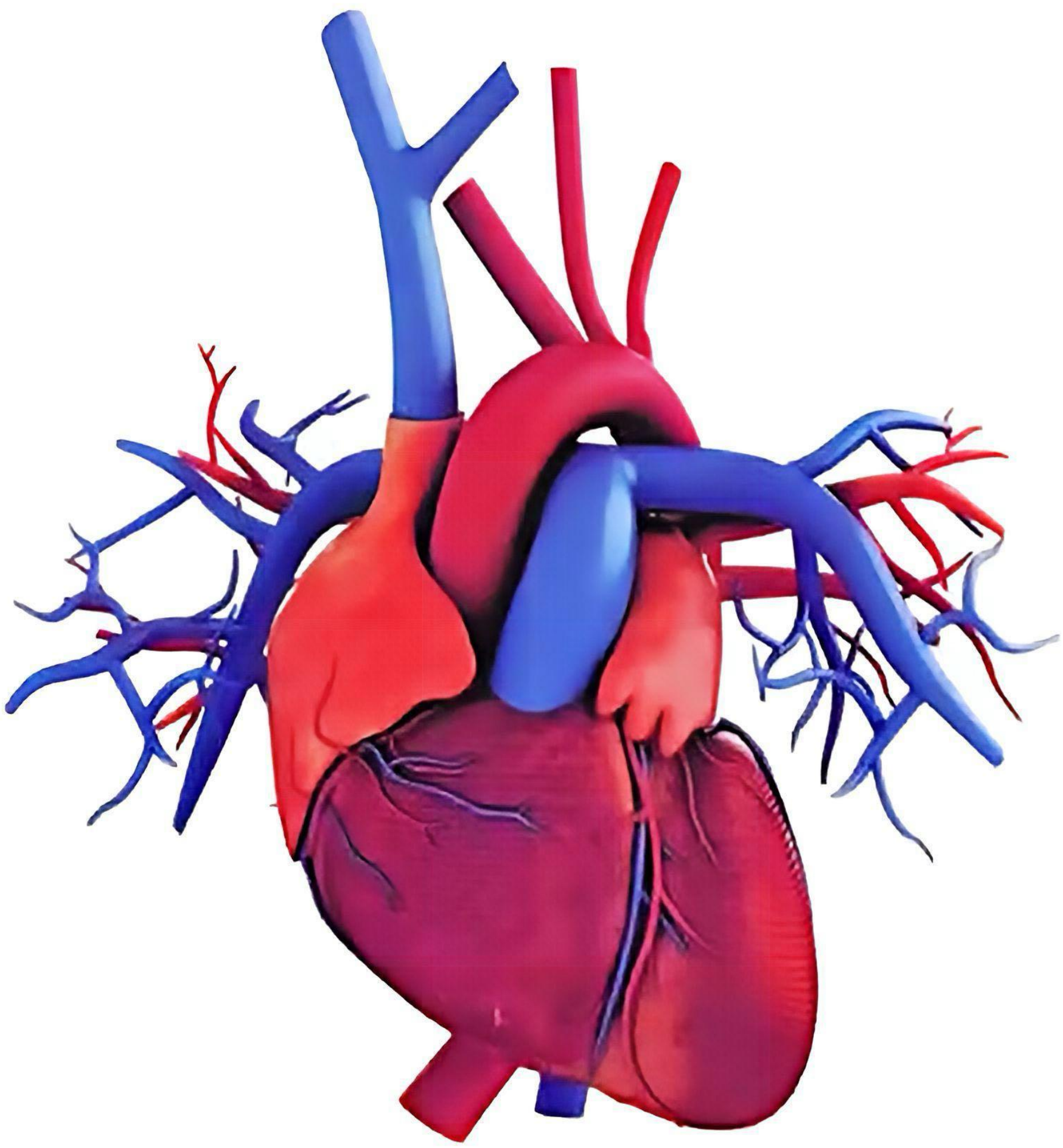

一、定义：

1、冠状动脉：它是供应心肌细胞营养的动脉，从主动脉根部起源，主要可分为左前降支、左回旋支以及右冠状动脉。

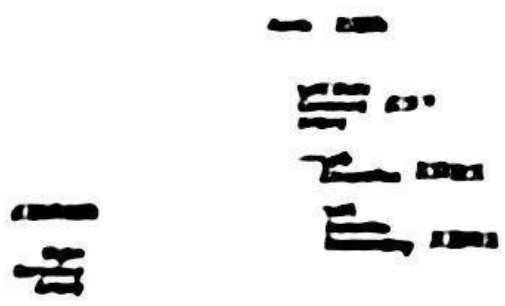

2、动脉粥样硬化：健康的动脉具有弹性，但随着时间推移、人体老化，动脉也会像长期使用的水管，变得斑驳老化，动脉壁会变硬，也就是动脉硬化。受累动脉的病变从内膜开始，局部有脂质积聚、纤维组织增生和钙质沉着，形成斑块，由于在动脉内膜积聚的脂质外观呈黄色粥样，因此称为动脉粥样硬化。

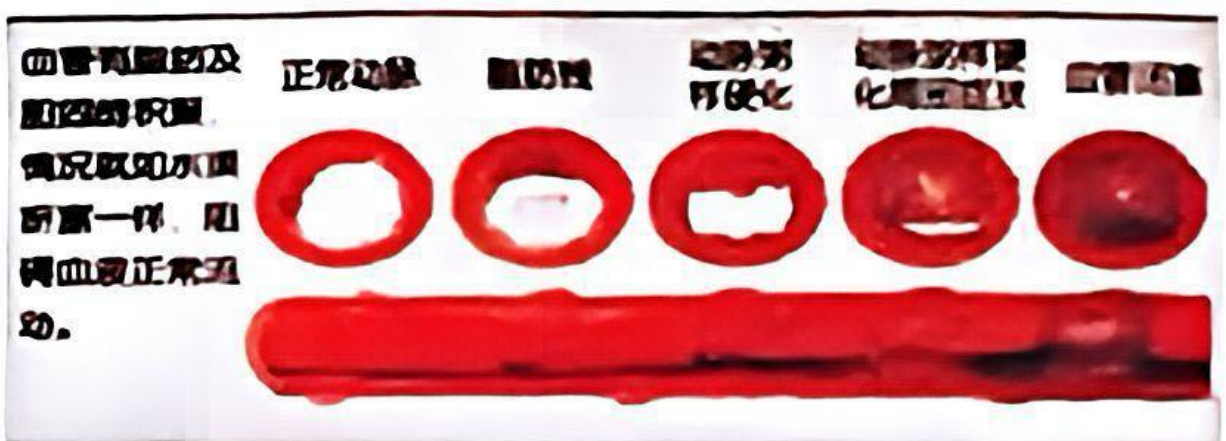

3、冠状动脉粥样硬化性心脏病：指冠状动脉发生粥样硬化引起管腔狭窄或闭塞，及冠脉循环功能改变，从而导致心肌缺血缺氧或者坏死而引起的心脏病，简称冠心病。2019年CCS指南重新分类为ACS急性冠脉综合征(不稳定性心绞痛、非ST段抬高型心梗及ST段抬高型心梗和CCS慢性冠脉综合征（无症状心肌缺血、

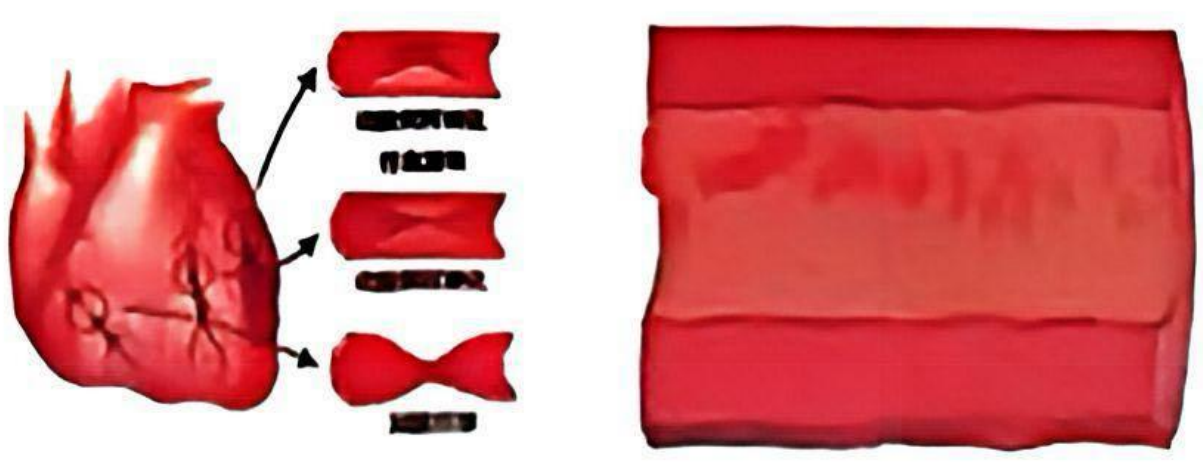

变的冠心病的不同发展阶段），强调冠心病是一个动脉粥样硬化斑块积累和冠脉功能改变的动态过程，具有相对稳定期，也可由于斑块破裂、斑块侵蚀及钙化结节等因素不稳定，强调了冠心病的动态性。

二、病因：

具体的病因尚未完全确定。但经研究表明，本病是多病因的疾病，即多种因素作用于不同的环节所致，这些因素称为危险因素。主要的危险因素有：

1.年龄、性别：40岁以上，男性的发病率比女性高.

2.血脂异常：血生化里总胆固醇、甘油三脂、低密度脂蛋白增高。

3.高血压。

4.吸烟：吸烟与不吸烟比较，发病率高了2-6倍。

5.糖尿病。

6.其他：肥胖、饮食习惯（常进食油炸等高热量食物）等。

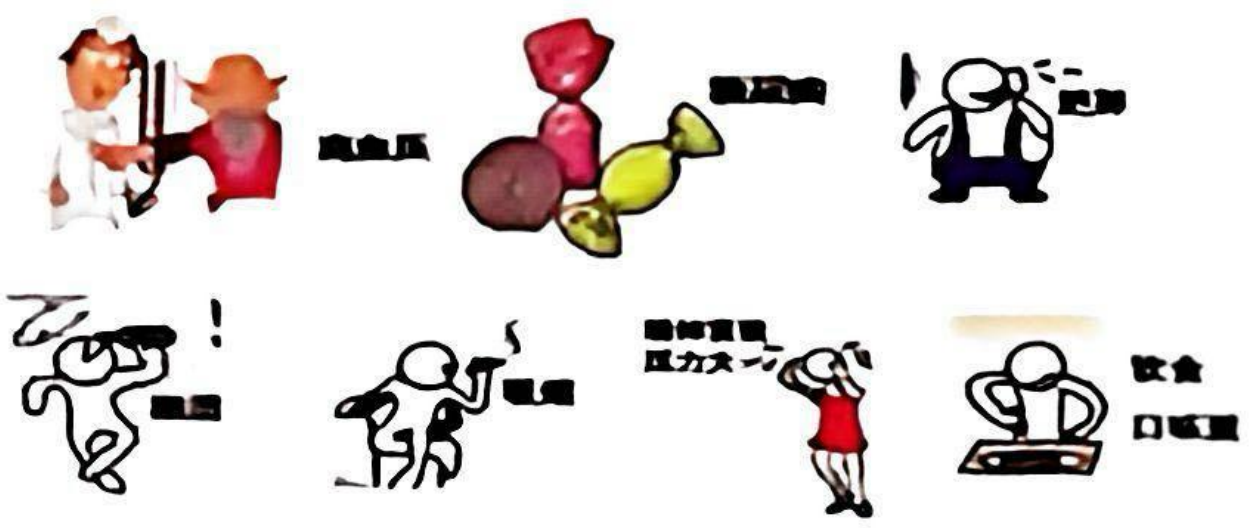

三、临床症状：

1.胸闷、胸痛，主要在胸骨体后，常放射至左肩、左臂内侧或至颈、下颌部等。

2.疼痛剧烈时可能会有恶心、呕吐、上腹部痛。

3.更厉害的会有坐立不安、脸色苍白、皮肤湿冷、反应迟钝、甚至晕倒。

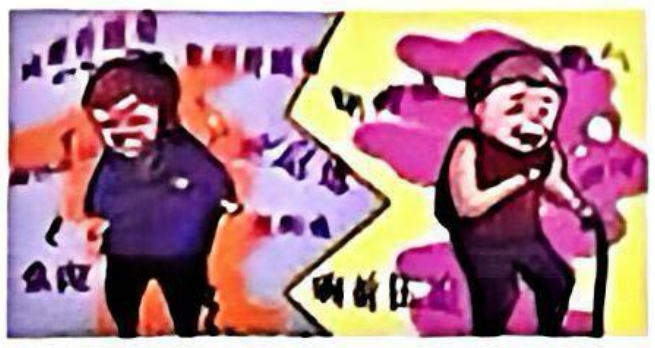

四、诱因：

1. 情绪激动，如愤怒、焦虑，过度兴奋。
2. 暴饮暴食。
3. 过重的体力劳动。
4. 寒冷等。

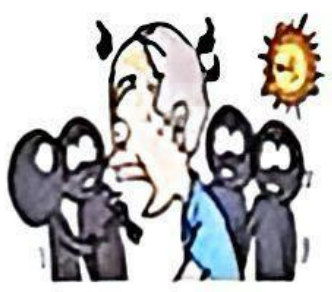
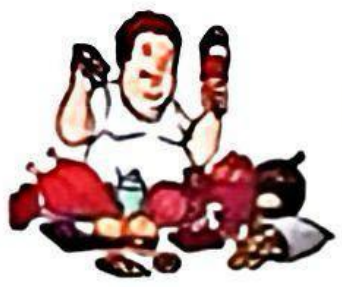
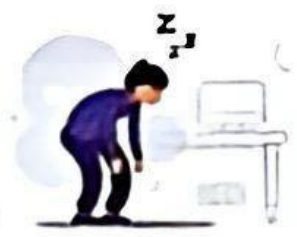
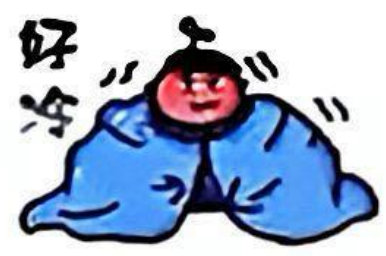

五、预防和治疗：

（一）行为和危险因素的调整。这是防治一体的措施。

1.控制体重：维持正常的体重，40岁以上者尤其应预防发胖。正常的体重简单计算法为：身高（厘米）-105=体重（公斤）；或者体重指数BMI=体重（公斤）/身高（米）^{2} 18.5~23.9为正常，可供参考。

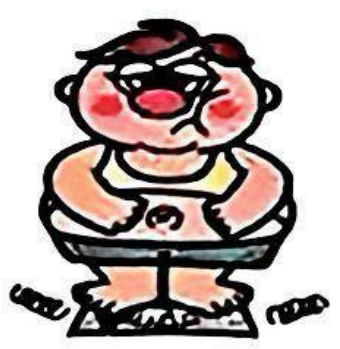

2.戒烟戒酒：烟草中的尼古丁、一氧化碳引发和加重动脉样硬化的发生和发展。酗酒或长期饮酒引起高脂血症。酒的热量高，多喝加重肥胖。

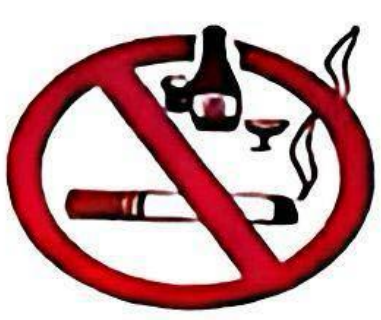

3.饮食：提倡清淡饮食，多吃富含维生素C（如新鲜蔬菜、瓜果）喝植物蛋白（如豆类及其制品）的食物。避免过饱。避免进食富含胆固醇的食物如：肥肉，奶油，蛋黄，动物内脏（肝、脑等），鱼子。

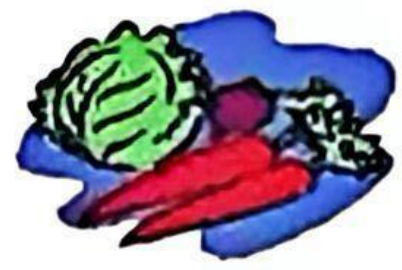

I25.103 1：冠状动脉粥样硬化性心脏病

4.规律活动：参加一定的体力劳动及体育活动，对预防肥胖、调整血脂都有好处，是预防本病的一项积极措施。但活动量要根据原来的身体情况来定。平时可适当参加体育活动如散步，慢跑，体操，太极拳，以不引起胸闷，胸痛，气急，为宜。尽量不要一个人去偏僻的地方。

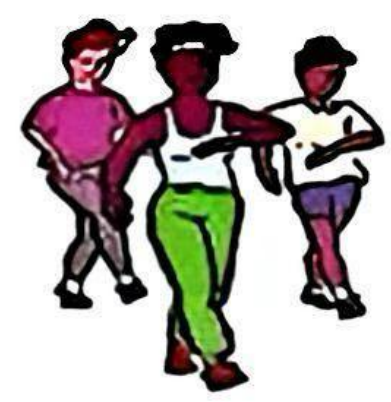
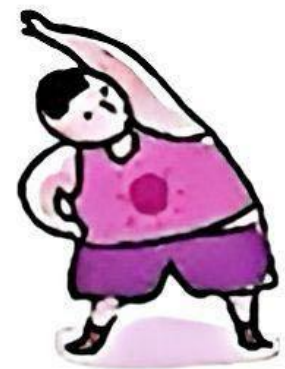
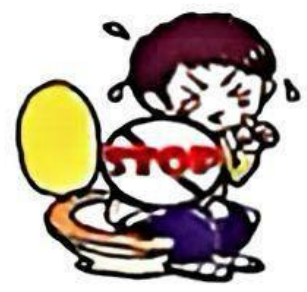

5.保持情绪稳定，切勿大喜大悲。保持大便通畅，必要时使用通便药物，切勿用力解大便！

6.积极的治疗与本病有关的一些疾病，如高血压、肥胖症、高脂血症、痛风、糖尿病等。

（二）内科药物治疗

1.抗血小板治疗：如阿司匹林、氯吡格雷、替格瑞洛等，可防止血栓形成。注意：长期服用此类药物的患者应随时注意药物可能存在的出血的危险，平时使用软毛牙刷、避免碰撞、避免粗纤维饮食。有牙龈出血、大便颜色褐色及时报告医生。（支架术后，需长期服药阿司匹林，氯吡格雷至少服用1年）。若有出血情况或其他不适请及时就医，不可随意停药，根据医嘱调整。）

2.改善心肌供血：可解除血管运动障碍，如硝酸酯类，β受体阻断剂，钙通道

滞剂等。注意：硝酸酯制剂有头痛，面红，心跳快等副作用，β受体阻滞剂会减慢心率（注意监测心率，心率控制在不低于60次/分，如有头晕不适，及时就诊）；钙通道阻滞剂副作用较小；若有上述症状，立即就医。

3. 调节血脂药：调整血脂，或者为了稳定血管壁上的杂质。注意：此类药物对于肝脏的影响比一般的药物要大，所以必须定期到医院化验肝功能。该类药物必须在医生的指导下服用，不可以自行断药，或者随意更改。

（三）介入治疗

心血管介入术可以明确冠状动脉狭窄的程度，并对狭窄血管进行介入治疗，恢复动脉的供血。

1. 冠脉造影及支架植入术：

那么到底是不是冠心病呢？我们需要通过一些检查来确诊，目前冠脉造影是诊断冠心病的金标准，它是一种创伤性检查，常规需要住院进行。

冠脉造影的穿刺途径：常见穿刺途径为桡动脉，当然也有通过股动脉穿刺进行操作。

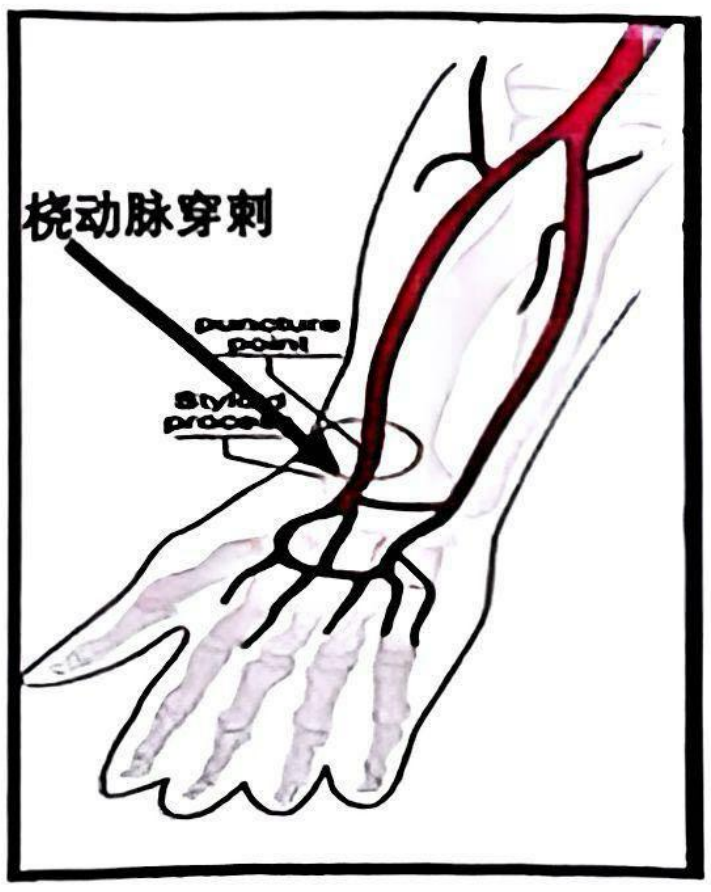

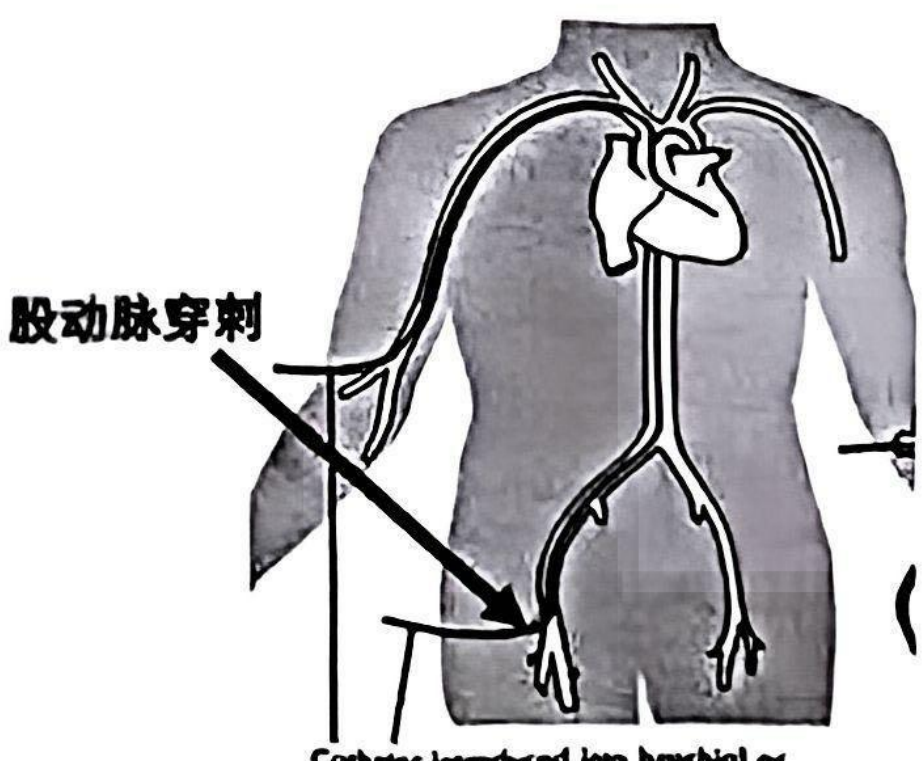

Catheter introduced into brachial or femoral artery and passed retrograde via aorta to left ventricle

2. 冠脉旋磨或准分子激光冠脉消融术：可作为钙化病变、CTO病变等的辅助治疗手段。

六、介入治疗注意事项：

术前准备

1.术前患者无需空腹，要求进食含水量少的食物，5分饱为宜。

2.术前请买好一次性拖鞋一双、一次性尿布中单一包，必要时备好尿壶或便盆、吸管等。

3.术日晨更换手术衣裤，不能穿内衣、内裤；去除首饰，假牙等物品。

4.手术前一天请配合护士留好留置针。

5.术前医生会完成知情同意谈话，请您落实手术费用，保证治疗的连续性。

术中配合

穿刺部位局部麻醉，在手术过程中患者是清醒的，只需听从指令，配合医生，如在操作过程中有胸闷胸痛等不适症状，及时告知医护人员。因病情不同，手术时间从几十分钟至几个小时不等，在此期间医生会让患者保持静卧。

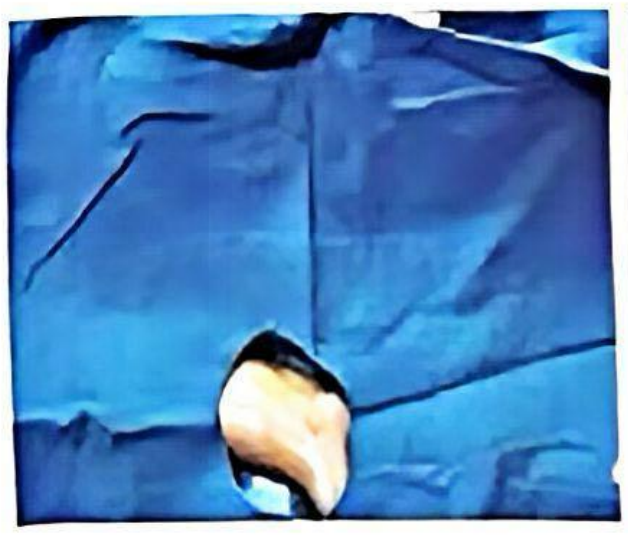

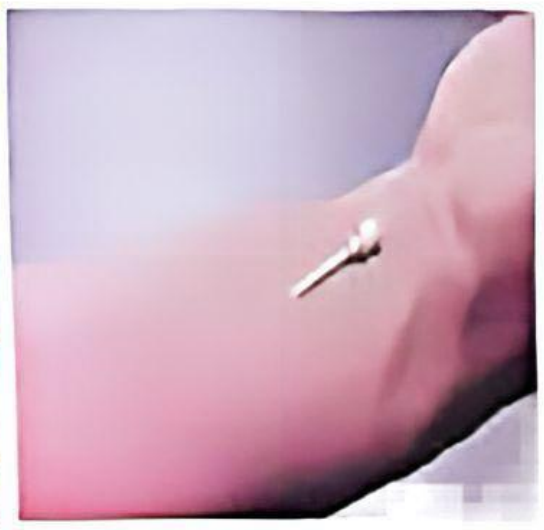

术后护理

1.伤口：术后穿刺侧伤口处会加压包扎，腕关节需制动，抬高肢体，需观察手指血运及局部皮肤温度、色泽、感觉，有无血肿，常规4小时后伤口无异常，会予松宽胶布；如止血夹，每2-3小时放松1次，无渗血情况4-6小时后完全放松，术后腕关节制动24小时，次日伤口换药。

I25.103 1：冠状动脉粥样硬化性心脏病

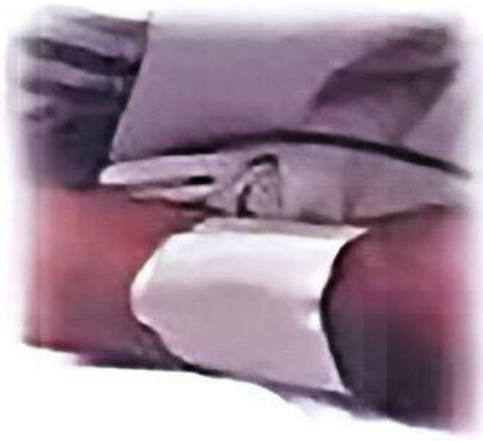

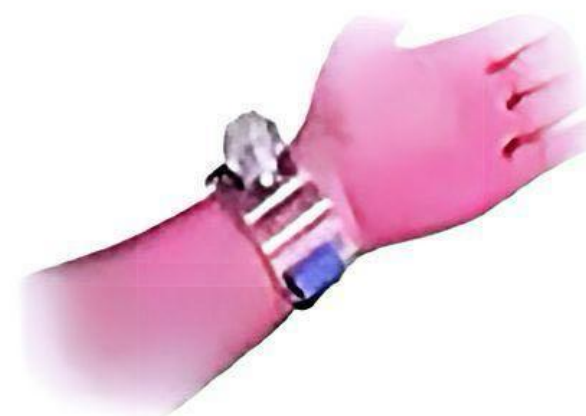

2.饮食：术后饮食应清淡低盐低脂，少量多餐，避免过饱。无心功能不全的患者术后6-8小时内鼓励多饮水，一般约1500-2000ml，分次服用，促进造影剂排泄。

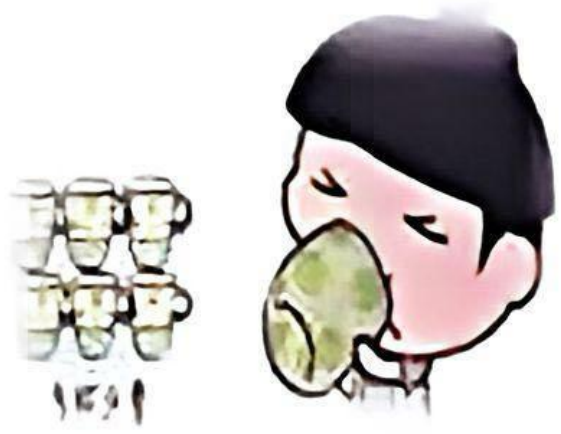

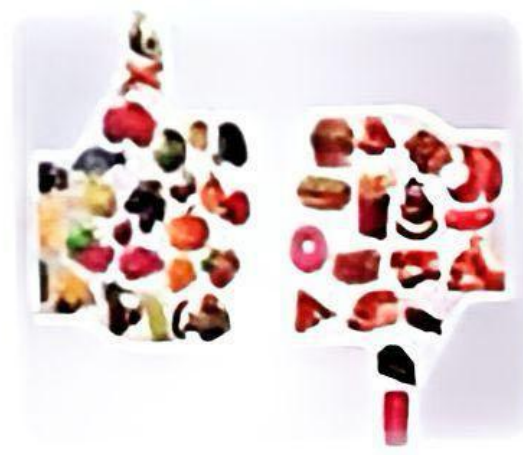

3.其他

1）术后常规给予心电、血氧饱和度及血压监测。

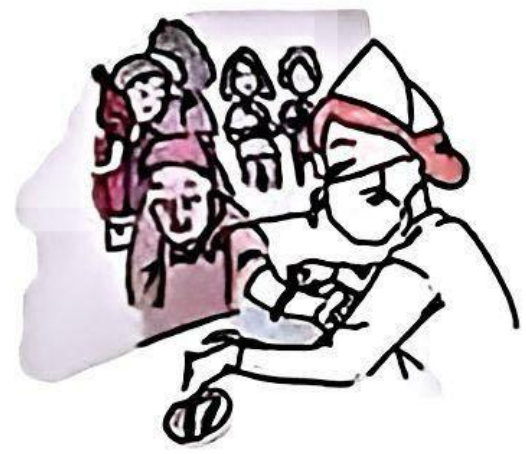

2）根据病情，配合医务人员完成术后心电图采集、抽血化验、用药等。

3）术后请家属陪护，拉上床栏，防止跌倒坠床，如有胸闷、出血等任何不适请随时告诉当班的医护人员。

七、外科治疗

有严重复杂的多支病变的患者，可能需至外科行冠脉搭桥术。

八、预后转归

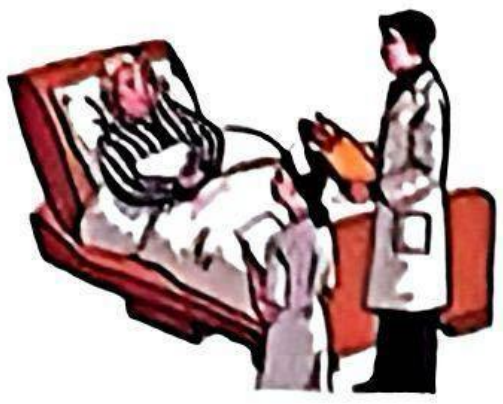

本病的预后与梗死范围的大小以及治疗是否及时有关。因此及早发现，及早住院显的尤为重要。

九、何时再来医院就诊？

1.门诊定期复诊，按医嘱复查血常规、血生化、凝血功能；心肌梗死患者还需复查心脏超声以了解心功能的恢复状况，复查动态心电图等了解心律失常情况。

2.自我监测：随身携带硝酸甘油或救心丸，出现胸闷胸痛时及时服用，及时就诊。如果发作的频率增加、程度加重、时间延长、诱发因素变化、硝酸类药物缓解作用减弱。及早拨打120或立即就医。

1. 卫生部2011版《临床护理实践指南》,人民军医出版社.
2. 尤黎明.内科护理学,第6版,北京:人民卫生出版社,2017:145-264.
3. 葛均波,徐永健.内科学,第8版,北京:人民卫生出版社,2013:155-346.
4. 张澍,霍勇.内科学.心血管内科分册,北京:人民卫生出版社,2016.
5. 2016中国经皮冠状动脉介入治疗指南.

修订日期：2023年

作者：蓝芳红

审校医生：周逸蒋

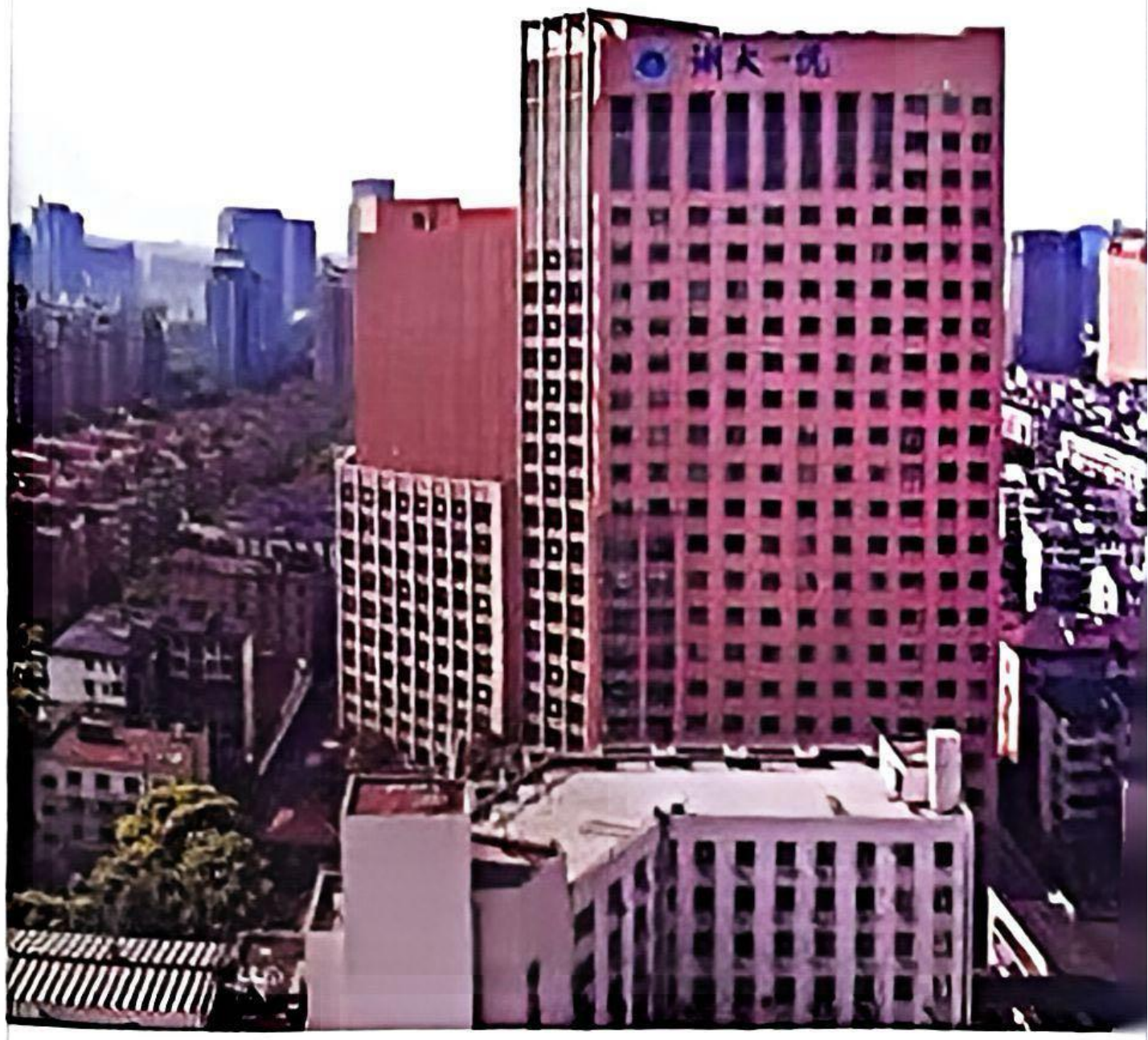

庆春院区地址： 杭州市庆春路79号（310003）
电话总机： 0571-87236114 0571-8723666
急诊电话： 0571-87236300
门诊电话： 0571-87236668
余杭院区地址： 杭州市余杭区文一西路1367号
急诊电话： 0571-87239955
门诊电话： 0571-87238001
之江院区地址： 杭州市西湖区梧桐路366号
急诊电话： 0571-87239909
门诊电话： 0571-87238158
城站院区地址： 杭州市上城区城站路58号
浙大一院官方网站： http://www.zy91.com
微信号： zdyyxczx
