# Supplementary material for: Effectiveness of a Behavior Change Wheel–Informed WeChat-Based Messaging Intervention to Improve Medication Adherence After Percutaneous Coronary Intervention: 12-Week Quasi-Experimental Study
Source: J Med Internet Res. 2026 Oct 7;28:e85192. doi: 10.2196/85192 (PMC13645199; doi:10.2196/85192)
Supplement: Supplementary file 2 [file jmir-v28-e85192-s001.docx]

**Multimedia Appendix 1. Pilot data on score changes from discharge to 1-month follow-up among participants in the intervention and control groups.**

**Table 1. Pilot Data on Changes in Scores From Discharge to 1-Month Follow-Up**

| **Group** | **ID** | **At Discharge** | **1-Month Follow-Up** | **Difference** |
| --- | --- | --- | --- | --- |
| Intervention (n=8) | 1 | 6.5 | 7.5 | 1.00 |
|  | 2 | 4.25 | 6.5 | 2.25 |
|  | 3 | 4.5 | 6.5 | 2.00 |
|  | 4 | 5.5 | 7.5 | 2.00 |
|  | 5 | 4.5 | 5.75 | 1.25 |
|  | 6 | 7.75 | 8.0 | 0.25 |
|  | 7 | 6.5 | 7.75 | 1.25 |
|  | 8 | 6.5 | 7.5 | 1.00 |
| Control (n=8) | 9 | 8.0 | 8.0 | 0.00 |
|  | 10 | 7.0 | 7.0 | 0.00 |
|  | 11 | 6.5 | 5.5 | -1.00 |
|  | 12 | 6.75 | 5.75 | -1.00 |
|  | 13 | 5.5 | 5.75 | 0.25 |
|  | 14 | 6.75 | 6.5 | -0.25 |
|  | 15 | 7.0 | 7.75 | 0.75 |
|  | 16 | 4.5 | 4.5 | 0.00 |
