# Supplementary material for: Effectiveness of a Behavior Change Wheel–Informed WeChat-Based Messaging Intervention to Improve Medication Adherence After Percutaneous Coronary Intervention: 12-Week Quasi-Experimental Study
Source: J Med Internet Res. 2026 Oct 7;28:e85192. doi: 10.2196/85192 (PMC13645199; doi:10.2196/85192)
Supplement: Supplementary file 3 [file jmir-v28-e85192-s003.docx]

**Application of BCW method to MedText-PCI**

# The present study applied BCW to systematically guide the development of MedText-PCI targeting medication adherence in post-PCI patients. The BCW is broadly split into three stages : (i) understanding the behavior; (ii) identification of intervention options; and (iii) identification of content and implementation options. Because mobile phones are widely used and text messaging is a common, low-cost, and effective tool to promote behavior change, text messaging was chosen as the delivery method at the start of intervention design. In this study, we expanded these into four practical stages to form the initial intervention plan (see Figure 1).

# Figure 1 Application of the BCW framework in this study

**Step 1. Behavioral Diagnosis**

Based on guideline recommendations and published evidence, the core behavior of interest was defined as daily intake of prescribed secondary prevention medications without missed or incorrect doses. To contextualize this behavior, we reviewed clinical literature, survey data, and expert consultations. Barriers included forgetfulness, concern about adverse effects, financial stress, and lack of family or physician support. Facilitators included strong risk perception, good health literacy, and regular follow-up. These determinants were categorized into capability, opportunity, and motivation according to the COM-B model.

**Step 2. Identify Intervention Functions and policy categories**

# Upon completing the behavioral diagnosis, we identified the intervention functions and policy categories most likely to induce change within the COM-B model. This process began by selecting suitable intervention functions (i.e., education, persuasion, incentivization, coercion, training, restriction, environmental restructuring, modeling, and enablement). The decision-making was guided by matrices in the BCW framework, which map COM-B components to potential intervention functions. These functions were then refined using the APEASE (Affordability, Practicability, Effectiveness/cost, Acceptability, Side-effects/Safety, and Equity) criteria, which help evaluate and select intervention components, policy categories, and BCTs suitable for inclusion. The selected intervention functions were added to the BCT intervention mapping tables (supplementary file 1). After finalizing the intervention functions, the matrix linking these functions to policy categories in the BCW was employed to identify appropriate policy categories (communication/marketing, guidelines, fiscal measures, regulation, legislation, environmental/social planning, and service provision). The APEASE criteria were again applied to further refine the selection.

**Step 3. Identify Content**

### After identifying the relevant intervention functions, we determined which BCTs would best support these functions in a text messaging intervention. The matrices within the BCW framework were used to guide the selection and to link BCTs to the chosen intervention functions. BCTs appropriate for each function are detailed in the BCW^[1]^. To systematically identify which BCTs were most likely to influence the target behaviors, we reviewed current literature.

After compiling the list of potentially relevant BCTs, each technique was reviewed using the APEASE framework to determine its feasibility and relevance. The assessment considered whether the technique could be implemented within reasonable resource limits, whether it was practical for large-scale delivery in the form of short text messages tailored to post-PCI patients, and whether its expected benefits justified the associated costs. In addition, attention was given to the likelihood that the selected techniques would be understandable and engaging for patients, with further acceptability testing planned in subsequent stages. Consideration was also given to the potential impact on health equity, aiming to ensure that the intervention would reduce rather than exacerbate disparities. Through this process, a final set of BCTs was established to guide the development of the text message library for improving medication adherence in post-PCI patients.

**Step 4. Message Development**

Message development was based on the previously identified BCTs. Drafts were prepared by ZX and WL, trained in BCT identification, and reviewed by FY and HZH, who have expertise in coronary heart disease and behavioral research. Each message was limited to 160 characters, the maximum supported by most mobile phones, to ensure accessibility for patients without smartphones, as smartphone-only interventions may increase health disparities. The wording was kept concise and free of jargon or abbreviations to reduce ambiguity. Readability was checked with the Flesch-Kincaid grade level test, and messages above the eighth-grade level were revised in line with recommendations for adult health communication. After these adjustments, professional reviewers confirmed clarity and accuracy.

For fidelity assessment, an independent reviewer (WWX), trained in BCT identification but not involved in drafting, coded all messages to verify consistency between intended and delivered techniques. Discrepancies were resolved through consensus discussions with WL, resulting in a finalized library in which each message accurately reflected the targeted BCTs.

**Step 1. Behavioral Diagnosis**

Based on guideline recommendations for post-PCI pharmacological management, we conducted a behavioral diagnosis under the guidance of the BCW framework to identify the final target behavior (Table 1) . Specifically, the behavior of interest was defined as taking medication daily without missing or incorrect doses. The target group for this behavior was post-PCI patients, who are at high risk for recurrent cardiovascular events if adherence is suboptimal. The behavior was specified to be performed every day after PCI, emphasizing consistency in medication intake as a critical component of secondary prevention. This behavior was expected to occur in various everyday contexts, including at home, at work, and within community settings. Detailed information on this target behavior specification is provided in Table 1. These specifications provided a clear and actionable foundation for the subsequent development of the text message intervention aimed at addressing the key barriers and facilitators related to medication adherence in this patient population. The identification of barriers and facilitators to engaging in the behavior, and their mapping onto the COM-B model, have been described in detail in our previous publication^[2]^.

### ****Table 1. Specification of the target behavior****

| **Target behavior** | **Medication non-adherence** |
| --- | --- |
| Actor | Post-PCI patients |
| Action | Taking medication daily without missing or incorrect doses |
| Time and frequency | Every day after PCI |
| Context | Home, workplace, community |

**Step 2. Identify Intervention Functions and policy categories**

All nine intervention functions and seven policy categories were initially identified as potentially relevant to address the barriers and facilitators related to medication adherence in post-PCI patients. Each of these candidate intervention options was evaluated using the APEASE criteria. As a result, six intervention functions (education, persuasion, training, incentivization, environmental restructuring, and enablement) were identified as appropriate for inclusion in a text messaging intervention for post-PCI patients. The remaining intervention functions were excluded because they were either inconsistent with the overall communication style suitable for post-PCI patients (restriction and coercion) or could not be effectively incorporated into brief text messages of less than 160 characters. The selected intervention functions were incorporated into the BCT mapping tables (supplementary 1). One policy category (communication/marketing) was identified as appropriate for implementation. The remaining policy categories (guidelines, environmental/social planning, legislation, service provision, regulation, and fiscal measures) were excluded because they were not feasible or applicable in this context (i.e., these categories could not be effectively targeted through text messaging delivery to this patient population).

**Step 3. Identify Content**

Through a literature review, we initially included a total of 60 BCTs (see Supplementary 2). After removing duplicates and screening for relevance, 45 potential BCTs were identified. These 45 BCTs were then evaluated using the APEASE criteria. Following this assessment, 30 BCTs were excluded. As a result, 15 BCTs were finally selected to address the identified barriers and facilitators in the text message content (Figure 2). These selected BCTs were subsequently incorporated into the BCW mapping tables (Supplementary 1).

**Figure 2 Diagram of BCTs identified in the literature and screened for inclusion.**

**Step 4. Message Development**

ZX and WL wrote the messages based on the 15 BCTs identified in the previous stage. An initial bank of 115 messages were written(evenly split into disease and medication information, benefits of adherence, and harms of non-adherence, (see Supplementary 3). All messages were written to correspond to a single BCT. All 115 messages were initially drafted by two medical experts and then revised by a communication specialist. The revisions mainly involved slight wording changes and punctuation adjustments to simplify the content and enhance the spirit of engagement and support. Messages were edited to maintain a reading level no higher than junior high school level.

After WWX completed the initial BCT coding, a consensus meeting was held between WWX and WL to finalize the BCT combinations assigned to each message. In total, 11 messages did not retain their originally intended BCTs (see Table 2), while 104 messages (90%) preserved their initial BCTs as planned.For the 11 messages where the original codes were not retained, at least one BCT was used for recoding to ensure conceptual consistency. Initially, '(1.7) Review outcome goal(s)' and '(4.1) Instruction on how to perform the behavior' were considered unsuitable for delivery via text messages, as ZX and WL believed that providing such instructions would be challenging to make acceptable or applicable to all participants. However, during the consensus process, some messages originally assigned to other BCTs were also coded as 4.1, and it was ultimately decided to include them. Overall, this iterative consensus process resulted in a finalized bank of 115 text messages designed to support medication adherence(see Supplementary 3).

**Table 2 Overview of behavior change technique alterations resulting from the fidelity check.**

| **Message Content** | **Initial Coding** | **Final Consensus Coding** |
| --- | --- | --- |
| Even if your blood lipid levels are normal, you still need to take statins. These drugs help stabilize plaques and prevent unexpected events. | (5.1) Information about health consequences | (15.1) Verbal persuasion about capability |
| Taking antiplatelet medications may cause bleeding. Always watch for gum, nasal, gastrointestinal bleeding (black stools), or blood in urine. Seek medical attention promptly if any occur. | (15.1) Verbal persuasion about capability | (5.1) Information about health consequences |
| Clinically, most patients who experience recurrence are those who do not adhere to their medication regimen. Patients who take medication as prescribed tend to remain stable. | (6.2) Social comparison | (5.1) Information about health consequences |
| Statins may affect liver function, so liver function tests should be performed regularly. | (5.1) Information about health consequences | (15.1) Verbal persuasion about capability |
| Attend regular follow-up visits, as your doctor needs to adjust your treatment plan based on your condition. | (1.4) Action planning | (8.1) Behavioral practice/rehearsal |
| Most patients report that sticking to medication has improved their symptoms. Think about what changes adherence has brought you. | (15.1) Verbal persuasion about capability | (6.2) Social comparison |
| Ask yourself: Has sticking to medication relieved my chest tightness or pain? | (15.3) Focus on past success | (15.4) Self-talk |
| According to research, nearly one-third of post-PCI patients have poor medication adherence. Not taking medication within the first year increases cardiovascular event risk by 40% and death risk by 34%. | (5.6) Information about emotional consequences | (5.1) Information about health consequences |
| Stopping medication can worsen coronary conditions and increase the risk of angina and reinfarction. | (5.6) Information about emotional consequences | (5.1) Information about health consequences |
| Taking medication helps you recover gradually, giving you more time and energy to spend with your family and friends. | (5.6) Information about emotional consequences | (5.1) Information about health consequences |
| According to WHO, in the first year after a heart attack, 10 out of 100 people die, and 8 of these deaths could be prevented by long-term medication adherence. | (5.6) Information about emotional consequences | (5.1) Information about health consequences |

**References**

[1] Michie S, Van Stralen MM, West R. The behavior change wheel: a new method for characterising and designing behavior change interventions. Implement Sci. 2011;6:42. doi:10.1186/1748-5908-6-42.

[2] Fang Y, Jiang Z, Han Z, Xiang X. Barriers and facilitators to medication adherence in patients after PCI surgery: a mixed-methods systematic review. Heart Lung. 2025;72:57-64. doi:10.1016/j.hrtlng.2025.03.008.

# supplementary 1

## English version

| **COM-B** | **Barriers and facilitators** | **Intervention function** | **Example BCTs** | **Example Message** |
| --- | --- | --- | --- | --- |
| Physical capability | Forgetfulness | Training | (1.2) Problem solving | Sometimes missing a dose is unavoidable, but don't be discouraged! More importantly, analyze the reasons, think about what went wrong, and prevent it from happening again. |
|  |  |  |  | If you miss a morning dose, make it up the same day; if not, do not take a double dose the next day. |
|  |  |  | (1.4) Action planning | If you often forget to take your medication, you can set an alarm or use a pillbox according to your preference. |
|  |  | Environmental restructuring | (7.1) Prompts/cues | Example: Mr./Ms. XX, please remember to take your medication on time. |
| Psychological capability | Lack of disease and medication knowledge | Education | (5.1) Information about health consequences | Why is regular follow-up necessary after stent implantation?   (1) Stent surgery only treats severely narrowed vessels; mild or moderate lesions still carry risks of angina or heart attack.   (2) Even after a successful procedure, there is still a risk of recurrence; the opened vessel may become narrowed again. |
|  |  | Training | (1.2) Problem solving | If acute chest pain occurs again:   (1) Stop activity and rest immediately.   (2) Take one nitroglycerin tablet under the tongue immediately. If ineffective, it may indicate an acute myocardial infarction—call emergency services and go to the hospital immediately. Even if relieved, seek medical evaluation as soon as possible. |
|  |  |  |  | If you are unsure about the purpose of your medications, refer to the PCI health manual, which has detailed information. |
|  |  | Persuasion | (15.1) Verbal persuasion about capability | Even if your blood lipid levels are normal, you still need to take statins. These drugs help stabilize plaques and prevent unexpected events. |
|  |  | Education | (5.1) Information about health consequences | Taking antiplatelet medications may cause bleeding. Always watch for gum, nasal, gastrointestinal bleeding (black stools), or blood in urine. Seek medical attention promptly if any of these occur. |
|  |  |  |  | Clinically, most patients who experience recurrence are those who do not adhere to their medication regimen. Patients who take medication as prescribed tend to remain stable, showing the importance of adherence. |
|  |  | Training | (4.1) Instruction on how to perform the behavior | Aspirin (antiplatelet agent)  Adverse reactions:  1 Gastrointestinal reactions, including nausea, vomiting, and upper abdominal discomfort.  2 Bleeding.  3 Allergic reactions, such as asthma, angioedema, or anaphylactic shock.  Precautions:  Take in the morning before meals to reduce gastric retention time and achieve maximum effectiveness. |
|  |  |  |  | Clopidogrel (Plavix)  Adverse reactions: Bleeding  Precautions:  1 Inform your doctor if you are using this medication before surgery.  2 Use with caution in patients with liver dysfunction.  3 Use with caution in patients with a bleeding tendency.  Additional notes: Take after breakfast in the morning. This medication may cause gastrointestinal mucosal damage. |
|  |  |  |  | Ticagrelor (Brilinta)  Adverse reactions: Bleeding  Precautions:  1 Inform your doctor if you are using this medication before surgery.  2 Use with caution in patients with liver dysfunction.  3 Use with caution in patients with a bleeding tendency. |
|  |  |  |  | Rivaroxaban (Xarelto)  Adverse reactions: Bleeding  Precautions:  1 Do not take with strong tea, beverages, or alcohol.  2 Use with caution in patients with a bleeding tendency. |
|  |  |  |  | Atorvastatin (Take after meals or before bedtime)  Effect: Lowers blood lipids and stabilizes plaques.  Precautions:  1 During treatment, cholesterol and creatine kinase levels should be checked regularly (monthly). Liver enzyme levels may increase during use; liver function tests should be monitored regularly.  2 Discontinue the medication if liver enzyme levels rise to three times the upper limit of normal, or if creatine kinase significantly increases, or if symptoms of myositis or pancreatitis occur. |
|  |  |  |  | Metoprolol (Betaloc) — a β-blocker  Effect: Lowers blood pressure, improves myocardial blood supply.  1 Reduces myocardial oxygen consumption by lowering myocardial contractility, heart rate, and blood pressure; prolongs diastole to improve coronary blood flow and perfusion, thereby reducing and relieving myocardial ischemia during daily activities or exercise, and improving quality of life.  2 Reduces infarct size, decreases fatal arrhythmias, and lowers acute phase mortality and cardiovascular event rates, including sudden cardiac death.  3 Long-term use improves long-term prognosis and increases survival rates. |
|  |  |  |  | Captopril  Effect: Lowers blood pressure.  Adverse reactions:  1 Rash, possibly accompanied by itching and fever, usually occurring within 4 weeks of treatment, appearing as maculopapular or urticarial rash; resolves with dose reduction, discontinuation, or antihistamines. 7%–10% may have eosinophilia or positive antinuclear antibodies.  2 Palpitations, tachycardia, chest pain.  3 Cough.  4 Altered taste. |
|  |  |  |  | Amlodipine (Norvasc)  Effect: Dilates peripheral arteries, relieves angina.  Adverse reactions:  1 Edema and headache.  2 Flushing and palpitations.  3 Hypotension.  Precautions: Monitor heart rate and blood pressure. |
|  |  |  |  | Isosorbide mononitrate (Imdur)  Effect: Dilates peripheral blood vessels.  Adverse reactions:  1 Headache, facial flushing.  2 Hypotension.  Precautions:  3 Can be taken whole or halved; do not chew or crush. |
|  |  |  |  | Bisoprolol (Concor) — a β-blocker  Effect: Lowers blood pressure and improves myocardial blood supply; used in the treatment of angina and myocardial infarction.  Adverse reactions: May include mild fatigue, chest tightness, dizziness, bradycardia, drowsiness, palpitations, headache, lower limb edema, diarrhea, constipation, nausea, abdominal pain, rash, itching, significant drop in blood pressure, slow pulse or atrioventricular block, tingling sensation or cold extremities, muscle weakness, painful muscle cramps, and reduced tearing. |
|  | Self-monitoring | Training | (2.3) Self-monitoring of behavior | Use a medication diary to evaluate your adherence. If there are missed doses, it means you need to work harder; if not, it shows you are doing great—please keep it up as it greatly benefits your health! |
|  | Integrating into daily habits | Training | (15.4) Self-talk | Link taking your medication with daily routines, such as asking yourself during morning brushing: Have I taken my medication today? |
|  |  |  | (7.1) Prompts/cues | Place your medication in a visible spot, such as on your bedside table. |
|  |  | Persuasion | (8.3) Habit formation | Take your medication at a fixed time every day. This helps you form a habit and makes it easier to remember. |
|  |  |  |  | Prepare your medications for the day when you get up, separating morning, noon, and evening doses and labeling them clearly to avoid forgetting. |
|  |  |  |  | Before and after each meal, ask yourself: Have I taken my medication? |
|  |  |  |  | By sticking to your medication routine, it will gradually become a habit. |
|  | Planning to overcome barriers | Environmental restructuring | (8.3) Habit formation | When going out, carry an emergency medicine kit (e.g., nitroglycerin). Also, do not forget to take aspirin and clopidogrel (or ticagrelor) after intervention surgery. |
|  |  |  |  | When traveling, prepare all your medications in advance and organize them into morning, noon, and evening sections in a pillbox. |
| Physical opportunity | Interference with daily life | Training | (1.4) Action planning | Life can be busy, and some people say work or travel causes them to forget medication. In this case, plan your medication schedule ahead of time and take it as planned. |
| Social opportunity | Regular follow-up | Training | (15.1) Verbal persuasion about capability | Statins may affect liver function, so liver function tests should be performed regularly. |
|  |  |  | (8.1)Behavioral practice/rehearsal | Attend regular follow-up visits, as your doctor needs to adjust your treatment plan based on your condition. |
|  | Social support | Training | (3.2) Social support (practical) | Sometimes you may forget to take your medication; think about who can remind and support you—perhaps your friends or family. |
|  |  |  |  | Many people find they are better able to adhere to medication under the supervision of family or friends. |
|  |  |  |  | There is an elderly patient, Mr. Wang, who is 75 years old. Five years after his stent surgery, he still adheres to his medication and follow-ups. If he can do it, so can you. |
|  |  |  |  | Your doctor wants you to share your feelings and any concerns so they can better help you. |
|  |  |  |  | If you have any questions about medications or other issues, contact us in time—we are happy to help you. |
|  |  |  | (15.1) Verbal persuasion about capability | Write down your medication questions and doubts, and give them to your doctor at your next visit. |
| Reflective motivation | Concerns about the future | Persuasion | (15.4) Self-talk | Remind yourself often that taking your medication helps you stay healthy and prevents disease progression. |
|  |  | Training | (15.1) Verbal persuasion about capability | Many patients say they worry about recurrence; the best way to prevent it is to adhere to your medication. |
|  |  | Enablement | (5.1) Information about health consequences | Medication can bring benefits, such as reducing hospitalizations and easing concerns about recurrence. |
|  | Perceived benefits and necessity | Persuasion | (15.1) Verbal persuasion about capability | Why continue medication after stent surgery? The stent only temporarily supports the treated vessel, and it does not treat other vessels. Medication helps prevent thrombosis in the stent and progression of atherosclerosis in other vessels. |
|  |  |  |  | Always remember: even if you feel better, do not stop your medication on your own! |
|  |  |  | (6.2) Social comparison | Most patients report that sticking to medication has improved their symptoms. Think about what changes adherence has brought you. |
|  |  |  | (15.4) Self-talk | Ask yourself: Has sticking to medication relieved my chest tightness or pain? |
|  |  |  | (5.1) Information about health consequences | According to research, nearly one-third of post-PCI patients have poor medication adherence. Not taking medication within the first year after PCI increases cardiovascular event risk by 40% and death risk by 34%. |
|  |  |  |  | Stopping medication can worsen coronary conditions and increase the risk of angina and reinfarction. |
|  |  |  |  | Taking medication helps you recover gradually, giving you more time and energy to spend with your family and friends. |
|  |  |  |  | According to WHO, in the first year after a heart attack, 10 out of 100 people die, and 8 of these deaths could be prevented by long-term medication adherence. |
|  |  |  | (9.2) Pros and cons | There may be many barriers to adherence, such as forgetfulness or side effects, but taking medication helps prevent recurrence. For your health, please stick to it! |
|  |  |  |  | What positive changes has medication adherence brought you? Fewer or no angina attacks? Improved test results? Feeling more energetic? |
|  | Self-efficacy | Persuasion | (1.1) Goal setting | Long-term adherence is challenging. Start with a small goal—stick to it for one week and give yourself a small reward when you succeed! |
|  |  | Education | (15.3) Focus on past success | You have stuck to it for 3 weeks. If you did not miss any doses, great job! If you did forget a few times, analyze the reasons and believe you can do better! |
|  |  |  |  | You have already persisted for 6 weeks. You can do it! Please keep it up—it's important for your condition. |
|  |  |  | (15.1) Verbal persuasion about capability | Understanding your treatment plan and medications is very important. Check the SMS you received in week 1 to review your medications, their effects, and side effects. |
|  |  | Persuasion | (1.7) Review outcome goal | Long-term adherence takes time. You may not have reached your goal yet, but you are on the right path. Keep going! |

## Chinese version

| **COM-B** | **障碍和促进因素** | **干预功能** | **BCT (CN)** | **短信** |
| --- | --- | --- | --- | --- |
| 身体能力 | 健忘 | 培训 | 解决问题 | 有时漏服药物是难以避免的，不要气馁!更重要的是分析原因，想一想哪里做的不到位，防止下次发生； |
|  |  |  |  | 若药物在早上发生漏服，则在当日补服；若当日未补服，切记不可在第二天补服。 |
|  |  |  | 行动计划 | 如果你忘记服药的情况时有发生，可以根据你的喜好采用设置一个服药闹钟、服药盒提醒等方法。 |
|  |  | 环境重构 | 提示/线索 | 示例：XX[先生/女士，请记得按时服用] |
| 心理能力 | 疾病药物知识缺乏 | 教育 | 关于健康后果的信息 | 支架术后为什么需要定期复查？ （1）支架手术仅处理高度狭窄的血管，轻中度病变的血管没有处理，仍有发生心绞痛或心肌梗死的危险； （2）支架手术虽然成功，但术后仍有一定的复发率，已经疏通的血管有再次狭窄的可能。 |
|  |  | 培训 | 解决问题 | 若急性胸痛再次发生： （1）停止活动，就地休息 （2）立即舌下含服硝酸甘油1片。如无效有可能发生了急性心肌梗死，应马上拨打急救电话，尽快去医院。如自行服药缓解，也应尽快去医院检查。 |
|  |  |  |  | 如果你不清楚你服用的药物的作用，打开PCI健康手册，里面有很详细的记录 |
|  |  | 说服 | 关于能力的口头说服 | 即使血脂正常也需要服用他汀类药物。这类药物可以稳定斑块，防止出现意外 |
|  |  | 教育 | 关于健康后果的信息 | 服用抗血小板药物可能导致出血，应时刻注意有无牙龈、鼻腔、消化道出血（黑便）、血尿等情况；若出现上述情况及时就医 |
|  |  |  |  | 临床上大多数复发的患者为不坚持服药的患者，而按时服药的患者病情相对稳定，可见遵医嘱服药的重要性 |
|  |  | 培训 | 关于如何执行一个行为的说明 | 您服用的药物：阿司匹林（抗血小板药） 不良反应： 1胃肠道反应，恶心、呕吐，上腹部不适 2出血 3过敏反应，哮喘，血管神经系统水肿或休克 注意事项：服用时间应为早上餐前服用，可减少药物在胃内的停留时间，发挥最大功效。 |
|  |  |  |  | 您服用的药物：氯吡格雷（波立维）： 不良反应：出血 注意事项： 1手术前使用该药应告知医生 2肝功能损害者慎用 3有出血倾向者慎用 注意事项：早上餐后服用，该药物具有胃肠道粘膜损害的副作用 |
|  |  |  |  | 您服用的药物：替格瑞洛（倍林达）： 不良反应：出血 注意事项： 1手术前使用该药应告知医生 2肝功能损害者慎用 3有出血倾向者慎用 |
|  |  |  |  | 您服用的药物：利伐沙班片（拜瑞妥） 不良反应：出血 注意事项： 1勿用浓茶，饮料，酒送服 2有出血倾向者慎用 |
|  |  |  |  | 您服用的药物：阿托伐他汀（餐后或睡前服用） 作用：降低血脂、稳定斑块 注意事项 1用药期间应定期（每个月）检查血胆固醇和血肌酸磷酸激酶。应用本品时血氨基转移酶可能增高，应定期监测肝功能试验。 2在本品治疗过程中如发生血氨基转移酶增高达正常高限的3倍，或血肌酸磷酸激酶显著增高或有肌炎、胰腺炎表现时，应停用本品。 |
|  |  |  |  | 您服用的药物：倍他乐克（美托洛尔）属于β受体阻滞剂 作用：降低血压、改善心肌供血 一是通过降低心肌收缩力、心率和血压, 使心肌耗氧量减少;同时延长心脏舒张期而增加冠脉及其侧支的血供和灌注, 从而减少和缓解日常活动或运动状态的心肌缺血发作, 提高生活质量。二是可缩小梗死范围, 减少致命性心律失常, 降低包括心脏性猝死在内的急性期病死率和各种心血管事件发生率。三是长期应用可改善患者的远期预后, 提高生存率。 |
|  |  |  |  | 您服用的药物：卡托普利： 作用:降低血压 不良反应： 1皮疹，可能伴有瘙痒和发热，常发生于治疗4周内，呈斑丘疹或荨麻疹，减量、停药或给抗组胺药后消失，7%～10%伴嗜酸性细胞增多或抗核抗体阳性。 2心悸，心动过速，胸痛 3咳嗽 4味觉迟钝 |
|  |  |  |  | 您服用的药物：洛活喜（苯硫磺氢氯地平片）： 作用：扩张外周动脉、缓解心绞痛 不良反应: 1、水肿和头痛 2、潮红、心悸 3、低血压 注意事项：监测心率和血压 |
|  |  |  |  | 您服用的药物：欣康片（单硝酸异山梨脂片）： 作用：扩张外周血管 不良反应： 1头痛、面部潮红 2低血压 注意事项： 3可整片或半片服用，不用嚼服或碾碎 |
|  |  |  |  | 您服用的药物：比索洛尔（康可）：属于β受体阻滞剂 作用：降低血压、改善心肌供血，用于心绞痛、心肌梗死的治疗。 不良反应：可见轻度乏力、胸闷、头晕、心动过缓、嗜睡、心悸、头痛和下肢水肿、腹泻、便秘、恶心、腹痛、红斑、瘙痒、血压明显下降、脉搏缓慢或房室传导阻滞、麻刺感或四肢冰凉、肌肉无力、肌肉痛性痉挛及泪少。 |
|  | 自我监测 | 培训 | 行为的自我监测 | 使用服药记录本去评估你的服药行为，如果存在漏服说明还需要努力，如果没有，说明你坚持得很好，请继续保持，这对你的疾病非常有益！ |
|  | 融入生活习惯 | 培训 | 自我对话 | 将服药和每天要做的事联系起来，比如早上刷牙时，问问自己，药物已经服用了吗？ |
|  |  |  | 提示/线索 | 把药物放在一个显眼的地方，比如床头柜上。 |
|  |  | 说服 | 形成习惯 | 每天在固定的时间服药，有助于您养成习惯，到时间就会想起来 |
|  |  |  |  | 每天起床提前把今天要服用的药物准备好，早中晚的药物放好并做好标识，到了服药的时候就不会忘记 |
|  |  |  |  | 在每次吃饭前、吃饭后，想一想，你的药已经吃过了吗？ |
|  |  |  |  | 坚持服药，慢慢地它会变成你的习惯。 |
|  | 做好应对障碍的计划 | 环境重构 | 解决问题 | 外出时应自备急救的药盒如硝酸甘油；另外介入术后服用的阿司匹林和氯吡格雷（替格瑞洛），千万不能忘记。 |
|  |  |  |  | 在外出旅游的时候，可以提前把药物都准备好，并放在服药盒，分为早、中、晚。 |
| 身体机会 | 日常生活的干扰 | 培训 | 行动计划 | 有时生活中会有许多事情，有的人说工作繁忙、外出旅游会导致忘记服药，针对这个情况可以提前计划好服药时间，时间到了就服药。 |
| 社会机会 | 定期随访 | 培训 | 关于能力的口头说服 | 他汀类药物可能会损害肝功能，故需定时复查肝功能指标。 |
|  |  |  | 行为形成 | 坚持定期随访，因为医生需要根据你的实际情况，调整药物治疗方案 |
|  | 社会支持 | 培训 | 社会支持(实际的) | 有时，你可能会忘记服药，想想谁能够经常给你提醒，给你提供帮助，可以是你的朋友或者你的家人。 |
|  |  |  |  | 许多人发现，在家人或朋友的监督下，自己更能坚持服药 |
|  |  |  |  | 有个老病人老王，他今年75岁，支架术后5年了仍然坚持规律服药和随访，他可以你也一定行 |
|  |  |  |  | 你的医生希望你告诉他们你的感受和你可能有的任何担忧，这样他们可更好地帮助你 |
|  |  |  |  | 有关药物或其他什么问题及时联系我们，我们非常乐意为你解答 |
|  |  |  | 关于口头能力的说服 | 写下你关于药物的问题和疑惑，下一次复查时交给医生。 |
| 反射性动机 | 对未来的担忧 | 说服 | 自我对话 | 经常告诉自己，坚持服药是为了让自己更健康，预防疾病恶化 |
|  |  | 培训 | 关于能力的口头说服 | 很多患者都说担心今后疾病复发，解决这最好的办法就是坚持服药 |
|  |  | 启发 | 提供健康益处的信息 | 药物能给你带来益处，比如说减少住院次数、让你不那么担心复发等 |
|  | 感知益处与必要性 | 说服 | 关于能力的口头说服 | 支架术后为何要继续服用药物？支架只能治好或者暂时支撑病变部位的血管，对其他血管没有治疗作用，目的在于防止支架内形成血栓、没放支架的血管动脉硬化继续恶化 |
|  |  |  |  | 时刻牢记，即使你自己感觉好多了，也不能擅自停药！ |
|  |  |  | 社会比较 | 大多数患者都说坚持服药让他们改善了，身体症状，想一想坚持服药给你带来了什么改变？ |
|  |  |  | 自我对话 | 问问自己，坚持服药是不是让我胸口闷，胸口疼的毛病缓解了？ |
|  |  |  | 关于健康后果的信息 | 据研究数据统计，近1/3的PCI术后患者药物依从性不佳，而PCI术后一年内不坚持服用药物会导致心血管事件风险增加40%，死亡风险增加34% |
|  |  |  |  | 如果停止服药，冠状动脉情况会更糟糕，心绞痛、再梗死的发生风险更高 |
|  |  |  |  | 服药可以帮助你慢慢恢复，让你有更多的时间和精力陪伴你的家人、朋友。 |
|  |  |  |  | 世界卫生组织数据显示，在心脏病发作后的第一年，100个人中就有10人死亡，其中8人可以通过长期坚持药物治疗挽救 |
|  |  |  | 利弊比较 | 坚持服药可能存在许多障碍比如说记性不好、服药后出现副作用，但它可以预防复发，因此坚持为了身体健康坚持服药吧！ |
|  |  |  |  | 坚持服药给您带来了哪些好的变化，是不是心绞痛发作次数减少甚至消失、各项指标趋向正常、感觉更有活力？ |
|  | 自我效能 | 说服 | 设定目标 | 长期坚持服药困难重重，可以从坚持服药1周这个小目标开始，完成了可以给自己一个小奖励！ |
|  |  | 教育 | 关注过去的成功 | 你已经坚持了3周，若你未发生漏服药物，那么你做的很棒，若你其中有几次忘记了服药，分析原因，相信你可以做的更好！ |
|  |  |  |  | 你已经坚持6周了，你可以做到，请继续保持，这对你病情稳定很重要！ |
|  |  |  | 关于能力的口头说服 | 了解自己的治疗方案和药物信息十分关键，看一看第1周收到的短信，你服用的药物有哪些以及它们的作用、副作用。 |
|  |  | 说服 | 回顾结果目标 | 长期坚持服药需要时间。可能你还没有达到你的目标，但你正在努力的过程中，继续保持！ |

# supplementary file 2

# BCT taxonomy for improving medication adherence (including intervention functions)

| **BCT（Chinese）** | **BCT(English)** | **Intervention Functions** |
| --- | --- | --- |
| 关于健康后果的信息 | (5.1) Information about health consequences | Education, Persuasion |
| 提供健康益处的信息 | (5.6) Information about health benefits | Education, Persuasion |
| 指导如何执行行为 | (4.1) Instruction on how to perform the behaviour | Education, Training |
| 信息关于先行事件 | (4.2) Information about antecedents | Education |
| 行动计划 | (1.4) Action planning | Training, Enablement |
| 设定行为目标 | (1.1) Goal setting (behaviour) | Training, Enablement |
| 回顾行为目标 | (1.5) Review behaviour goal(s) | Training, Enablement |
| 问题解决 | (1.2) Problem solving | Training, Enablement |
| 行为契约 | (1.8) Behavioral contract | Enablement, Persuasion |
| 承诺 | (1.9) Commitment | Enablement, Persuasion |
| 比较利弊 | (9.2) Pros and cons | Persuasion, Education |
| 提示/线索 | (7.1) Prompts/cues | Environmental restructuring, Enablement |
| 减少提示 | (7.3) Reduce prompts/cues | Environmental restructuring |
| 重塑物理环境 | (12.1) Restructuring the physical environment | Environmental restructuring |
| 重塑社会环境 | (12.2) Restructuring the social environment | Environmental restructuring |
| 添加环境对象 | (12.5) Adding objects to the environment | Environmental restructuring |
| 行为形成 | (8.1) Behavioural practice/rehearsal | Training |
| 行为替代 | (8.2) Behaviour substitution | Training, Enablement |
| 形成习惯 | (8.3) Habit formation | Training, Enablement |
| 生动展示行为 | (6.1) Demonstration of the behaviour | Modelling |
| 社会比较 | (6.2) Social comparison | Persuasion, Modelling |
| 他人批准信息 | (6.3) Information about others’ approval | Persuasion |
| 自我监测行为 | (2.3) Self-monitoring of behaviour | Enablement |
| 监测行为结果 | (2.7) Feedback on outcome(s) of behaviour | Enablement |
| 反馈行为 | (2.2) Feedback on behaviour | Enablement |
| 生物反馈 | (2.6) Biofeedback | Enablement |
| 生理/健康监测 | (2.5) Monitoring outcome(s) by others | Enablement |
| 社会支持（实际的） | (3.2) Social support (practical) | Enablement |
| 社会支持（非特指） | (3.1) Social support (unspecified) | Enablement |
| 口头能力说服 | (15.1) Verbal persuasion about capability | Persuasion |
| 心理排练 | (15.2) Mental rehearsal of successful performance | Training |
| 聚焦过去成功 | (15.3) Focus on past success | Persuasion |
| 自我对话 | (15.4) Self-talk | Persuasion |
| 身份识别塑造 | (13.1) Identification of self as role model | Modelling |
| 重构信念 | (13.2) Framing/reframing | Persuasion |
| 不兼容信念 | (13.3) Incompatible beliefs | Persuasion |
| 重视身份认同 | (13.4) Valued self-identity | Persuasion |
| 行为相关身份 | (13.5) Identity associated with changed behaviour | Persuasion |
| 社会奖励 | (10.4) Social reward | Incentivisation |
| 自我奖励 | (10.9) Self-reward | Incentivisation |
| 物质奖励 | (10.1) Material incentive (behaviour) | Incentivisation |
| 社会激励 | (10.5) Social incentive | Incentivisation |
| 非特指激励 | (10.6) Non-specific incentive | Incentivisation |
| 自我激励 | (10.7) Self-incentive | Incentivisation |
| 行为结果激励 | (10.8) Incentive (outcome) | Incentivisation |
| 情景特定奖励 | (10.6) Situation-specific reward | Incentivisation |
| 删除奖励 | (14.9) Reduce reward frequency | Coercion |
| 情境代价 | (14.1) Behaviour cost | Coercion |
| 惩罚 | (14.2) Punishment | Coercion |
| 奖励近似 | (14.4) Reward approximation | Incentivisation |
| 奖励完成 | (14.5) Rewarding completion | Incentivisation |
| 惩罚移除 | (14.10) Remove punishment | Coercion |
| 身体变化 | (12.6) Body changes | Environmental restructuring |
| 想象惩罚 | (16.1) Imaginary punishment | Persuasion |
| 想象奖励 | (16.2) Imaginary reward | Persuasion |
| 替代行为奖励 | (14.8) Reward alternative behaviour | Incentivisation |
| 非兼容行为奖励 | (14.7) Reward incompatible behavior | Incentivisation |
| 情绪后果信息 | (5.6) Information about emotional consequences | Persuasion |
| 预期后悔 | (5.5) Anticipated regret | Persuasion |
| 降低负面情绪 | (11.2) Reduce negative emotions | Enablement |

# supplementary 3 SMS message library

**English version**

| **No.** | **Message Content** |
| --- | --- |
| 1 | Why do you need regular follow-up after stent implantation? (1) The stent only treats severely narrowed arteries. Mild or moderate lesions remain untreated and can still cause angina or myocardial infarction. (2) Even if the procedure is successful, there is still a risk of restenosis in the treated arteries. |
| 2 | If acute chest pain occurs again: (1) Stop activity and rest immediately. (2) Take one nitroglycerin tablet sublingually. If ineffective, it may indicate an acute myocardial infarction. Call emergency services immediately and go to the hospital as soon as possible. Even if relieved, still go to the hospital for evaluation. |
| 3 | If you are unsure about the purpose of your medications, check your PCI health handbook for detailed information. |
| 4 | Even if your lipid levels are normal, you still need to take statins. These medications help stabilize plaques and prevent unexpected events. |
| 5 | Taking antiplatelet drugs may cause bleeding. Watch for gum bleeding, nosebleeds, black stools, or blood in the urine. Seek medical attention promptly if these occur. |
| 6 | Most patients who relapse do so because they fail to take their medications consistently. Patients who take medications as prescribed generally have more stable conditions. This highlights the importance of adherence. |
| 7 | Your medication: Aspirin (antiplatelet drug) Adverse effects:  1. Gastrointestinal reactions: nausea, vomiting, upper abdominal discomfort 2. Bleeding 3. Allergic reactions: asthma, angioedema, or shock Instructions: Take in the morning before meals to reduce gastric retention time and maximize efficacy. |
| 8 | Your medication: Clopidogrel (Plavix) Adverse effects: Bleeding Instructions: 1. Inform your doctor before surgery if using this drug. 2. Use cautiously in patients with liver dysfunction. 3. Use cautiously in patients with bleeding tendencies. Take after breakfast as it may irritate the gastrointestinal mucosa. |
| 9 | Your medication: Ticagrelor (Brilinta) Adverse effects: Bleeding Instructions: 1. Inform your doctor before surgery if using this drug. 2. Use cautiously in patients with liver dysfunction. 3. Use cautiously in patients with bleeding tendencies. |
| 10 | Your medication: Rivaroxaban (Xarelto) Adverse effects: Bleeding Instructions: 1. Do not take with strong tea, beverages, or alcohol. 2. Use cautiously in patients with bleeding tendencies. |
| 11 | Your medication: Atorvastatin (take after meals or at bedtime) Function: Lowers lipids, stabilizes plaques Instructions: 1. Regularly monitor cholesterol and creatine kinase levels (monthly). Liver enzymes may increase, so liver function tests are needed. 2. Discontinue if liver enzymes increase to 3 times the upper limit of normal, or if creatine kinase significantly increases, or in case of myositis or pancreatitis symptoms. |
| 12 | Your medication: Metoprolol (Betaloc), a β-blocker Function: Lowers blood pressure, improves myocardial blood supply It reduces heart rate and contractility, lowering myocardial oxygen demand. It prolongs diastole, improving coronary perfusion, thus reducing ischemic episodes and improving quality of life. It also reduces infarct size, decreases fatal arrhythmias, lowers mortality, and improves long-term prognosis. |
| 13 | Your medication: Captopril Function: Lowers blood pressure Adverse effects: 1. Rash (may itch or have fever, often within 4 weeks), usually resolves after dose reduction, discontinuation, or antihistamines. 2. Palpitations, tachycardia, chest pain 3. Cough 4. Taste disturbances. |
| 14 | Your medication: Amlodipine (Norvasc) Function: Dilates peripheral arteries, relieves angina Adverse effects: 1. Edema and headache 2. Flushing, palpitations 3. Hypotension Instructions: Monitor heart rate and blood pressure. |
| 15 | Your medication: Isosorbide mononitrate (Imdur) Function: Dilates peripheral vessels Adverse effects: 1. Headache, facial flushing 2. Hypotension Instructions: Swallow whole or half tablet; do not chew or crush. |
| 16 | Your medication: Bisoprolol (Concor), a β-blocker Function: Lowers blood pressure, improves myocardial blood supply; used for angina and myocardial infarction Adverse effects: Mild fatigue, chest tightness, dizziness, bradycardia, drowsiness, palpitations, headache, leg edema, diarrhea, constipation, nausea, abdominal pain, rash, pruritus, significant hypotension, slow pulse or AV block, tingling or cold limbs, muscle weakness or cramps, and dry eyes. |
| 17 | Statins may impair liver function; regular liver function tests are required. |
| 18 | Regular follow-ups are necessary so your doctor can adjust your medication plan based on your condition. |
| 19 | Remind yourself that taking medication consistently keeps you healthier and prevents disease progression. |
| 20 | Many patients worry about recurrence. The best way to prevent it is to adhere to your medication regimen. |
| 21 | Medications bring benefits, such as fewer hospitalizations and less worry about recurrence. |
| 22 | Why continue medications after stent placement? Stents only treat the affected vessel segment and do not address other vessels. Medications prevent thrombosis inside the stent and further atherosclerosis. |
| 23 | Always remember: never stop medications on your own, even if you feel better! |
| 24 | Most patients report symptom improvement after adherence. Reflect on the positive changes medication has brought you. |
| 25 | Ask yourself: Has adherence reduced or relieved your chest tightness and pain? |
| 26 | Data shows that nearly one-third of PCI patients have poor adherence. Stopping medication in the first year increases cardiovascular event risk by 40% and death risk by 34%. |
| 27 | Stopping medications worsens coronary artery disease, increasing risks of angina and reinfarction. |
| 28 | Medication helps you gradually recover, giving you more time and energy to spend with family and friends. |
| 29 | WHO data show that 10 out of 100 heart attack patients die within the first year, but 8 of them could be saved with long-term medication. |
| 30 | Although adherence can be difficult due to poor memory or side effects, it is essential for preventing recurrence and maintaining health. |
| 31 | What positive changes have you noticed? Fewer angina attacks, normal lab results, more energy? |
| 32 | Use a medication logbook to evaluate adherence. Missed doses mean improvement is needed; if none are missed, continue — it greatly benefits your condition. |
| 33 | Long-term adherence is challenging. Start with a one-week goal and reward yourself when achieved. |
| 34 | Link medication to daily routines, such as asking yourself after brushing teeth if you've taken your medicine. |
| 35 | Place medications in visible spots, like on the nightstand. |
| 36 | Take medications at a fixed time each day to develop a habit. |
| 37 | Prepare your medications in advance each morning for each dose and label clearly to avoid forgetting. |
| 38 | Before or after meals, ask yourself if you've taken your medication. |
| 39 | Over time, taking medication will become a habit. |
| 40 | If you often forget, think about who can remind and support you — family or friends. |
| 41 | Many people find it easier to adhere when supervised by family or friends. |
| 42 | Mr. Wang, 75 years old, has taken medications consistently for five years after PCI. You can do it too! |
| 43 | Tell your doctor about your feelings and concerns so they can help you better. |
| 44 | Contact us for any medication or other questions. We are happy to help. |
| 45 | Write down medication-related questions and give them to your doctor at your next visit. |
| 46 | Carry an emergency kit when going out, including nitroglycerin. Never forget aspirin and clopidogrel (or ticagrelor) after PCI. |
| 47 | When traveling, prepare and sort your medications in advance (morning, noon, evening) in a pillbox. |
| 48 | Missing doses is sometimes unavoidable. Don't be discouraged! Analyze the reasons and avoid next time. |
| 49 | If you miss a morning dose, take it the same day. Do not double up the next day. |
| 50 | If you frequently forget, consider setting medication alarms or using a pillbox reminder. |
| 51 | Busy work or travel can lead to missed doses. Plan ahead and take medication on time. |
| 52 | You've persisted for 3 weeks! If no missed doses, great job! If a few misses, analyze and improve. |
| 53 | You've persisted for 6 weeks! Keep going — it's important for stability. |
| 54 | Understanding your treatment and medications is crucial. Review the week 1 messages to recall your medications and their effects. |
| 55 | Long-term adherence takes time. You may not have reached your goal yet, but keep going! |
| 56 | Example: Mr./Ms. XX, please remember to take your medication on time. |
| 57 | Though not easy, every dose is an investment in your health. |
| 58 | Reviewing the positive changes from adherence can motivate you to continue. |
| 59 | Imagine fewer hospital stays and more time with family if you adhere. |
| 60 | If adherence feels overwhelming, focus on just completing today. |
| 61 | Reward yourself after several days without missing doses. |
| 62 | If side effects occur, consult your doctor — never stop on your own. |
| 63 | Create a reminder plan with family or friends to support you. |
| 64 | For travel, pre-sort medications into small boxes for convenience. |
| 65 | Choose a favorite song as a medication alarm to make it fun. |
| 66 | Reflect each night if you took medications properly and rate yourself. |
| 67 | Record feelings and symptom changes to strengthen adherence. |
| 68 | Place a reminder card at home saying 'Have you taken your medicine today?'. |
| 69 | Medication is self-care and self-responsibility. |
| 70 | Share your adherence experience with your doctor for support and encouragement. |
| 71 | Draw a smiley on the calendar each week you complete; more smiles, more achievement. |
| 72 | Plan daily medication times and link to routines (e.g., after breakfast). |
| 73 | Seeing stabilized blood pressure or lipids boosts confidence. |
| 74 | If setbacks occur, don’t give up; reflect and restart. |
| 75 | Imagine your condition worsening if you stop, as self-warning. |
| 76 | Talk to fellow patients for mutual encouragement. |
| 77 | Taking medications for your family is an act of love and responsibility. |
| 78 | Adherence benefits both today and future health. |
| 79 | Treat medication as a 'daily task' to enjoy the satisfaction of completion. |
| 80 | Silently say 'This is for my health' when taking medicine. |
| 81 | Keep water and medications by your bed for morning convenience. |
| 82 | Tell your doctor about your adherence during visits to show your efforts. |
| 83 | Feeling more energetic is the best proof — keep going! |
| 84 | Imagine your healthier, more secure self after one year of adherence. |
| 85 | When feeling weak, recall the satisfaction of past adherence. |
| 86 | Adherence reassures your family and reduces their worry. |
| 87 | Discuss your medication plan with family; their support strengthens you. |
| 88 | For business trips, prepare a checklist and pack medications in advance. |
| 89 | Adherence is the best support for your doctor’s treatment plan. |
| 90 | It can also reduce hospitalization costs and overall medical expenses. |
| 91 | Review your weekly adherence and set new goals for the next week. |
| 92 | Remind yourself 'I can do it' to reinforce confidence. |
| 93 | The more you understand about your medications, the easier it is to adhere. |
| 94 | If you miss a dose, don’t blame yourself. Adjust and keep going. |
| 95 | Imagine your future self thanking your current self for today's efforts. |
| 96 | Use different colored labels to distinguish multiple medications and avoid confusion. |
| 97 | If you need to take medication at night, prepare it in advance by the bed. |
| 98 | When your family sees your efforts, they will cheer you on. |
| 99 | Take photos to document your adherence journey. |
| 100 | Record improvements in a 'gratitude diary' to reinforce positive feelings. |
| 101 | Create a reward system and treat yourself after achieving milestones. |
| 102 | Pack medications in a small bag for easy access anytime. |
| 103 | Review goals and effects with your doctor regularly to stay motivated. |
| 104 | When facing challenges, review your adherence journey to regain strength. |
| 105 | Adherence reduces unexpected events such as heart attacks and rehospitalizations. |
| 106 | After each dose, say silently, 'I'm cheering for my health.' |
| 107 | Adherence cultivates patience and willpower. |
| 108 | Make medication a natural habit, like eating or washing your face. |
| 109 | Long-term adherence allows for more enjoyment of life. |
| 110 | Share your experience to inspire and help others. |
| 111 | Adherence is the best gift for your family. |
| 112 | Taking medication can set an example for others. |
| 113 | Set daily reminders on your phone for convenience. |
| 114 | Imagine your future self being grateful for your persistence today. |
| 115 | Adherence brings you closer to 'health freedom' — it is truly worth it. |

**Chinese version**

| **序号** | **短信内容** |
| --- | --- |
| 1 | 支架术后为什么需要定期复查？ （1）支架手术仅处理高度狭窄的血管，轻中度病变的血管没有处理，仍有发生心绞痛或心肌梗死的危险； （2）支架手术虽然成功，但术后仍有一定的复发率，已经疏通的血管有再次狭窄的可能。 |
| 2 | 若急性胸痛再次发生： （1）停止活动，就地休息 （2）立即舌下含服硝酸甘油1片。如无效有可能发生了急性心肌梗死，应马上拨打急救电话，尽快去医院。如自行服药缓解，也应尽快去医院检查。 |
| 3 | 如果你不清楚你服用的药物的作用，打开PCI健康手册，里面有很详细的记录 |
| 4 | 即使血脂正常也需要服用他汀类药物。这类药物可以稳定斑块，防止出现意外 |
| 5 | 服用抗血小板药物可能导致出血，应时刻注意有无牙龈、鼻腔、消化道出血（黑便）、血尿等情况；若出现上述情况及时就医 |
| 6 | 临床上大多数复发的患者为不坚持服药的患者，而按时服药的患者病情相对稳定，可见遵医嘱服药的重要性 |
| 7 | 您服用的药物：阿司匹林（抗血小板药） 不良反应： 1胃肠道反应，恶心、呕吐，上腹部不适 2出血 3过敏反应，哮喘，血管神经系统水肿或休克 注意事项：服用时间应为早上餐前服用，可减少药物在胃内的停留时间，发挥最大功效。 |
| 8 | 您服用的药物：氯吡格雷（波立维）： 不良反应：出血 注意事项： 1手术前使用该药应告知医生 2肝功能损害者慎用 3有出血倾向者慎用 注意事项：早上餐后服用，该药物具有胃肠道粘膜损害的副作用 |
| 9 | 您服用的药物：替格瑞洛（倍林达）： 不良反应：出血 注意事项： 1手术前使用该药应告知医生 2肝功能损害者慎用 3有出血倾向者慎用 |
| 10 | 您服用的药物：利伐沙班片（拜瑞妥） 不良反应：出血 注意事项： 1勿用浓茶，饮料，酒送服 2有出血倾向者慎用 |
| 11 | 您服用的药物：阿托伐他汀（餐后或睡前服用） 作用：降低血脂、稳定斑块 注意事项 1用药期间应定期（每个月）检查血胆固醇和血肌酸磷酸激酶。应用本品时血氨基转移酶可能增高，应定期监测肝功能试验。 2在本品治疗过程中如发生血氨基转移酶增高达正常高限的3倍，或血肌酸磷酸激酶显著增高或有肌炎、胰腺炎表现时，应停用本品。 |
| 12 | 您服用的药物：倍他乐克（美托洛尔）属于β受体阻滞剂 作用：降低血压、改善心肌供血 一是通过降低心肌收缩力、心率和血压, 使心肌耗氧量减少;同时延长心脏舒张期而增加冠脉及其侧支的血供和灌注, 从而减少和缓解日常活动或运动状态的心肌缺血发作, 提高生活质量。 二是可缩小梗死范围, 减少致命性心律失常, 降低包括心脏性猝死在内的急性期病死率和各种心血管事件发生率。 三是长期应用可改善患者的远期预后, 提高生存率。 |
| 13 | 您服用的药物：卡托普利： 作用:降低血压 不良反应： 1皮疹，可能伴有瘙痒和发热，常发生于治疗4周内，呈斑丘疹或荨麻疹，减量、停药或给抗组胺药后消失，7%～10%伴嗜酸性细胞增多或抗核抗体阳性。 2心悸，心动过速，胸痛 3咳嗽 4味觉迟钝 |
| 14 | 您服用的药物：洛活喜（苯磺氢氯地平片）： 作用：扩张外周动脉、缓解心绞痛 不良反应: 1、水肿和头痛 2、潮红、心悸 3、低血压 注意事项：监测心率和血压 |
| 15 | 您服用的药物：欣康片（单硝酸异山梨脂片）： 作用：扩张外周血管 不良反应： 1头痛、面部潮红 2低血压 注意事项： 3可整片或半片服用，不用嚼服或碾碎 |
| 16 | 您服用的药物：比索洛尔（康可）：属于β受体阻滞剂 作用：降低血压、改善心肌供血，用于心绞痛、心肌梗死的治疗。 不良反应：可见轻度乏力、胸闷、头晕、心动过缓、嗜睡、心悸、头痛和下肢水肿、腹泻、便秘、恶心、腹痛、红斑、瘙痒、血压明显下降、脉搏缓慢或房室传导阻滞、麻刺感或四肢冰凉、肌肉无力、肌肉痛性痉挛及泪少。 |
| 17 | 他汀类药物可能会损害肝功能，故需定时复查肝功能指标。 |
| 18 | 坚持定期随访，因为医生需要根据你的实际情况，调整药物治疗方案 |
| 19 | 经常告诉自己，坚持服药是为了让自己更健康，预防疾病恶化 |
| 20 | 很多患者都说担心今后疾病复发，解决这最好的办法就是坚持服药 |
| 21 | 药物能给你带来益处，比如说减少住院次数、让你不那么担心复发等 |
| 22 | 支架术后为何要继续服用药物？支架只能治好或者暂时支撑病变部位的血管，对其他血管没有治疗作用，目的在于防止支架内形成血栓、没放支架的血管动脉硬化继续恶化 |
| 23 | 时刻牢记，即使你自己感觉好多了，也不能擅自停药！ |
| 24 | 大多数患者都说坚持服药让他们改善了，身体症状，想一想坚持服药给你带来了什么改变？ |
| 25 | 问问自己，坚持服药是不是让我胸口闷，胸口疼的毛病缓解了？ |
| 26 | 据研究数据统计，近1/3的PCI术后患者药物依从性不佳，而PCI术后一年内不坚持服用药物会导致心血管事件风险增加40%，死亡风险增加34% |
| 27 | 如果停止服药，冠状动脉情况会更糟糕，心绞痛、再梗死的发生风险更高 |
| 28 | 服药可以帮助你慢慢恢复，让你有更多的时间和精力陪伴你的家人、朋友。 |
| 29 | 世界卫生组织数据显示，在心脏病发作后的第一年，100个人中就有10人死亡，其中8人可以通过长期坚持药物治疗挽救 |
| 30 | 坚持服药可能存在许多障碍比如说记性不好、服药后出现副作用，但它可以预防复发，因此坚持为了身体健康坚持服药吧！ |
| 31 | 坚持服药给您带来了哪些好的变化，是不是心绞痛发作次数减少甚至消失、各项指标趋向正常、感觉更有活力？ |
| 32 | 使用服药记录本去评估你的服药行为，如果存在漏服说明还需要努力，如果没有，说明你坚持得很好，请继续保持，这对你的疾病非常有益！ |
| 33 | 长期坚持服药困难重重，可以从坚持服药1周这个小目标开始，完成了可以给自己一个小奖励！ |
| 34 | 将服药和每天要做的事联系起来，比如早上刷牙时，问问自己，药物已经服用了吗？ |
| 35 | 把药物放在一个显眼的地方，比如床头柜上。 |
| 36 | 每天在固定的时间服药，有助于您养成习惯，到时间就会想起来 |
| 37 | 每天起床提前把今天要服用的药物准备好，早中晚的药物放好并做好标识，到了服药的时候就不会忘记 |
| 38 | 在每次吃饭前、吃饭后，想一想，你的药已经吃过了吗？ |
| 39 | 坚持服药，慢慢地它会变成你的习惯。 |
| 40 | 有时，你可能会忘记服药，想想谁能够经常给你提醒，给你提供帮助，可以是你的朋友或者你的家人。 |
| 41 | 许多人发现，在家人或朋友的监督下，自己更能坚持服药 |
| 42 | 有个老病人老王，他今年75岁，支架术后5年了仍然坚持规律服药和随访，他可以你也一定行 |
| 43 | 你的医生希望你告诉他们你的感受和你可能有的任何担忧，这样他们可更好地帮助你 |
| 44 | 有关药物或其他什么问题及时联系我们，我们非常乐意为你解答 |
| 45 | 写下你关于药物的问题和疑惑，下一次复查时交给医生。 |
| 46 | 外出时应自备急救的药盒如硝酸甘油；另外介入术后服用的阿司匹林和氯吡格雷（替格瑞洛），千万不能忘记。 |
| 47 | 在外出旅游的时候，可以提前把药物都准备好，并放在服药盒，分为早、中、晚。 |
| 48 | 有时漏服药物是难以避免的，不要气馁!更重要的是分析原因，想一想哪里做的不到位，防止下次发生； |
| 49 | 若药物在早上发生漏服，则在当日补服；若当日未补服，切记不可在第二天补服。 |
| 50 | 如果你忘记服药的情况时有发生，可以根据你的喜好采用设置一个服药闹钟、服药盒提醒等方法。 |
| 51 | 有时生活中会有许多事情，有的人说工作繁忙、外出旅游会导致忘记服药，针对这个情况可以提前计划好服药时间，时间到了就服药。 |
| 52 | 你已经坚持了3周，若你未发生漏服药物，那么你做的很棒，若你其中有几次忘记了服药，分析原因，相信你可以做的更好！ |
| 53 | 你已经坚持6周了，你可以做到，请继续保持，这对你病情稳定很重要！ |
| 54 | 了解自己的治疗方案和药物信息十分关键，看一看第1周收到的短信，你服用的药物有哪些以及它们的作用、副作用。 |
| 55 | 长期坚持服药需要时间。可能你还没有达到你的目标，但你正在努力的过程中，继续保持！ |
| 56 | 示例：XX[先生/女士，请记得按时服用] |
| 57 | 坚持服药虽然不易，但每次坚持都是对健康的投资。 |
| 58 | 回顾一下过去坚持服药带来的积极变化，会让你更有动力继续坚持。 |
| 59 | 想象未来如果坚持服药，你会更少住院、更多时间陪伴家人。 |
| 60 | 如果感觉服药负担大，可以尝试一次只关注完成一天的目标。 |
| 61 | 如果连续几天都没有忘记服药，可以给自己一些小奖励，比如一个小礼物。 |
| 62 | 如果有副作用困扰，及时与医生沟通，不要擅自停药。 |
| 63 | 和朋友或家人一起制定服药提醒计划，让他们也成为你的监督者。 |
| 64 | 如果旅途中携带药物不方便，可以提前分装到小药盒中，方便随身携带。 |
| 65 | 选择一首喜欢的歌作为服药的提示铃声，增加坚持的乐趣。 |
| 66 | 每天晚上回顾一下当天是否已正确服药，并为自己打分。 |
| 67 | 记录服药后感觉的变化，例如精神状态或症状改善，帮助增强坚持的信心。 |
| 68 | 在家里明显的位置贴一张「今天吃药了吗？」的提醒卡片。 |
| 69 | 服药是一种照顾自己的方式，是对自己负责的表现。 |
| 70 | 告诉医生你的坚持经历，他们会为你点赞，也可能会给出更多支持。 |
| 71 | 每完成一周服药，就在日历上画一个小笑脸，累计的笑脸越多，成就感越大。 |
| 72 | 计划好每天服药的时间，最好固定与日常活动（如早餐后）相结合。 |
| 73 | 发现服药后血压、血脂等指标稳定，会让你更有信心。 |
| 74 | 如果中途出现挫折，不要气馁，及时总结并重新开始。 |
| 75 | 想象如果一直不服药，疾病可能会加重，这是一种自我警示。 |
| 76 | 可以和同样经历的病友交流经验，互相鼓励共同坚持。 |
| 77 | 为家人和爱的人坚持服药，是对他们的责任和爱。 |
| 78 | 坚持服药不仅为了今天，更是为了未来健康的每一天。 |
| 79 | 如果觉得麻烦，可以把服药想象成「每日任务」，完成后享受「打卡」的成就感。 |
| 80 | 每次拿药时，默念「这是为了我的健康」。 |
| 81 | 在床头准备水和药物，方便早晨第一时间服用。 |
| 82 | 每次复诊时，告诉医生你坚持服药的情况，让医生知道你的努力。 |
| 83 | 如果服药让你感觉更有活力，那就是最好的证明，坚持下去吧！ |
| 84 | 想象完成一年服药计划后，自己会更有安全感、更健康。 |
| 85 | 当觉得无力时，回忆之前坚持服药成功时的满足感。 |
| 86 | 坚持服药能让家人更安心，减少他们的担心。 |
| 87 | 和家人讨论你的服药计划，他们的理解与参与会让你更有力量。 |
| 88 | 如果出差或旅行前觉得麻烦，提前写好清单，一次性准备好药物。 |
| 89 | 坚持服药其实也是对医生治疗方案的最大配合。 |
| 90 | 坚持用药也可以减少住院的经济负担，降低整体医疗费用。 |
| 91 | 每周末总结一周的服药情况，为下周制定新的目标。 |
| 92 | 每一次提醒自己「我做得到」，帮助强化信心。 |
| 93 | 了解更多药物知识，越了解越能坚持。 |
| 94 | 若偶尔漏服，不要责怪自己，重要的是立即调整，持续前进。 |
| 95 | 想象未来的自己感谢现在努力坚持的自己。 |
| 96 | 如果同时服用多种药物，可以给每种药贴上不同颜色的小标签，避免混淆。 |
| 97 | 如果夜里需要服药，可提前准备好水和药放在床头柜。 |
| 98 | 当家人看到你的努力，也会为你加油打气。 |
| 99 | 坚持服药时，可以拍照记录，形成「坚持相册」。 |
| 100 | 如果服药后症状好转，可记录「感谢日记」强化好感受。 |
| 101 | 设计自己的「服药奖励机制」，每坚持一段时间就犒劳自己一次。 |
| 102 | 可以把药物装在小包里，随时随地都能方便服用。 |
| 103 | 定期和医生回顾服药目标和效果，保持对进展的期待。 |
| 104 | 当遇到困难时，回顾之前坚持的历程，重新获得力量。 |
| 105 | 坚持服药帮助减少意外事件的发生，如心梗、再次住院等。 |
| 106 | 每次完成服药后，可以默默说一句「我为健康加油」。 |
| 107 | 坚持不仅改变了身体，更能培养耐心和意志力。 |
| 108 | 让服药成为一种生活习惯，就像吃饭、洗脸一样自然。 |
| 109 | 如果长期保持，未来会有更多享受美好生活的机会。 |
| 110 | 和朋友分享坚持经验，也许能帮助更多人。 |
| 111 | 坚持服药是对家人最好的礼物。 |
| 112 | 服药不仅是个人行动，也可以成为影响周围人的榜样。 |
| 113 | 可以在手机备忘录里设置每日提醒，更精准方便。 |
| 114 | 想象多年后，身体依然健康的自己，感谢今天坚持不懈的努力。 |
| 115 | 坚持服药让你离「健康自由」更近一步，这是最值得的坚持。 |
