# Supplementary material for: Effectiveness of a Behavior Change Wheel–Informed WeChat-Based Messaging Intervention to Improve Medication Adherence After Percutaneous Coronary Intervention: 12-Week Quasi-Experimental Study
Source: J Med Internet Res. 2026 Oct 7;28:e85192. doi: 10.2196/85192 (PMC13645199; doi:10.2196/85192)
Supplement: Supplementary file 4 [file jmir-v28-e85192-s004.docx]

**Table 1.** WeChat Text-Messaging Delivery Plan

| **Category** | **Stage**  **(Weeks)** | **Primary COM-B Focus** | **Key Topics** | **Message IDs**  **(from library)** |
| --- | --- | --- | --- | --- |
| **Reminder messages** | Discharge–12 weeks | Prompting (cue to action) | Short directive reminders delivered ~30 minutes before each usual dosing time, based on baseline regimen. Example: “Please remember to take your morning aspirin.” | System-generated, not from message library |
| **Non-reminder messages** | Stage 1 (Weeks 1–3) | Capability | PCI and medication knowledge; rationale for follow-up; safe use and adverse-effect management; self-monitoring and logging | 1, 3–5, 7–18, 21–23, 32, 66–67, 73 |
|  | Stage 2 (Weeks 4–6) | Motivation | Benefits of adherence; behavioral feedback and positive reinforcement; reducing intentional nonadherence; self-efficacy and emotional support | 19–22, 26–31, 57–61, 78–81, 83–86, 90, 93–95, 101–105, 111–115 |
|  | Stage 3 (Weeks 7–12) | Opportunity | Habit formation and environmental cues; travel/busy-day planning; missed-dose handling; self-reward; family, clinician, and peer support | Habit/cues: 33–39, 71–72, 79, 108; Travel/missed-dose: 47–51, 64, 88; Rewards: 61, 101; Social support: 40–44, 63, 87–89, 98 |

**Footnote.** Message IDs correspond to the standardized bilingual text-messaging library developed for this study and provided in Multimedia Appendix 3.
