# Supplementary material for: Effectiveness of a Behavior Change Wheel–Informed WeChat-Based Messaging Intervention to Improve Medication Adherence After Percutaneous Coronary Intervention: 12-Week Quasi-Experimental Study
Source: J Med Internet Res. 2026 Oct 7;28:e85192. doi: 10.2196/85192 (PMC13645199; doi:10.2196/85192)
Supplement: Supplementary file 5 [file jmir-v28-e85192-s005.docx]

**Appendix 5. Diagnostic Assessment of ANCOVA Assumptions Using Observed Data**

Supplementary Table S1. Diagnostic Assessment of ANCOVA Assumptions Using Observed Data

| Outcome | Group × baseline P | Quadratic term P | JB P | BP P | Analytical response |
| --- | --- | --- | --- | --- | --- |
| MMAS-8 | <.001 | .960 | .018 | <.001 | Interaction term + HC3 |
| BMQ–Necessity | .063 | .100 | .582 | .031 | HC3 |
| BMQ–Concerns | .991 | .334 | .486 | .292 | HC3 |
| BMQ–N–C Differential | .146 | .529 | .419 | .077 | HC3 |
| SEAMS | .273 | .560 | <.001 | .002 | HC3 |
| SAQ–Physical limitation | .939 | .659 | .073 | <.001 | HC3 |
| SAQ–Angina stability | .094 | .156 | .683 | .285 | HC3 |
| SAQ–Angina frequency | .034 | .378 | .903 | .084 | Interaction term + HC3 |
| SAQ–Treatment satisfaction | .605 | .619 | .030 | .128 | HC3 |
| SAQ–Quality of life | .832 | .885 | .345 | .755 | HC3 |
| SAQ–Summary score | .747 | .204 | <.001 | .150 | HC3 |

Note: Homogeneity of regression slopes was assessed using the group-by-baseline interaction, and linearity was assessed using a quadratic baseline term. Residual normality and homoscedasticity were assessed using the Jarque–Bera and Breusch–Pagan tests, respectively. Group-by-baseline interactions were retained for MMAS-8 and SAQ–Angina frequency. HC3 heteroscedasticity-robust standard errors were used for all final models. JB indicates Jarque–Bera; BP, Breusch–Pagan; HC3, heteroscedasticity-robust covariance estimator.
