# Supplementary material for: Effectiveness of a Behavior Change Wheel–Informed WeChat-Based Messaging Intervention to Improve Medication Adherence After Percutaneous Coronary Intervention: 12-Week Quasi-Experimental Study
Source: J Med Internet Res. 2026 Oct 7;28:e85192. doi: 10.2196/85192 (PMC13645199; doi:10.2196/85192)
Supplement: Supplementary file 6 [file jmir-v28-e85192-s006.docx]

**Appendix 6. Results of the Sensitivity Analyses**

**Table A 6.1. Per-protocol sensitivity analysis of outcomes at 12 weeks**

| Outcome | Intervention group (n=44) | Control group (n=43) | Adjusted mean difference (95% CI) | P value |
| --- | --- | --- | --- | --- |
|  | Adjusted mean (SE), 95% CI | Adjusted mean (SE), 95% CI |  |  |
| Primary outcome | | | | |
| MMAS-8 score | 7.39 (0.05), [7.30–7.49] | 6.26 (0.10), [6.06–6.45] | 1.14 [0.92 to 1.36] | <.001 |
| Secondary outcomes | | | | |
| Beliefs About Medicines Questionnaire | | | | |
| BMQ–Necessity | 22.44 (0.18), [22.08–22.80] | 20.87 (0.23), [20.43–21.32] | 1.57 [0.99 to 2.14] | <.001 |
| BMQ–Concerns | 12.11 (0.24), [11.62–12.59] | 15.31 (0.21), [14.90–15.72] | -3.20 [-3.84 to -2.57] | <.001 |
| BMQ–Necessity–Concerns Differential | 10.40 (0.37), [9.65–11.14] | 5.50 (0.34), [4.83–6.17] | 4.89 [3.90 to 5.89] | <.001 |
| Self-Efficacy for Appropriate Medication Use | | | | |
| SEAMS | 28.13 (0.40), [27.33–28.93] | 24.15 (0.16), [23.82–24.47] | 3.98 [3.12 to 4.84] | <.001 |
| Seattle Angina Questionnaire | | | | |
| SAQ–Physical limitation | 85.73 (1.08), [83.59–87.87] | 77.75 (0.51), [76.74–78.76] | 7.98 [5.58 to 10.38] | <.001 |
| SAQ–Angina stability | 73.24 (2.03), [69.21–77.28] | 60.52 (2.16), [56.22–64.82] | 12.72 [6.82 to 18.63] | <.001 |
| SAQ–Angina frequency | 93.29 (0.68), [91.95–94.64] | 85.61 (1.11), [83.41–87.81] | 7.68 [5.10 to 10.26] | <.001 |
| SAQ–Treatment satisfaction | 83.92 (0.83), [82.27–85.58] | 83.48 (0.84), [81.81–85.15] | 0.44 [-1.93 to 2.82] | .710 |
| SAQ–Quality of life | 71.41 (1.39), [68.65–74.17] | 64.33 (1.27), [61.80–66.86] | 7.08 [3.27 to 10.88] | <.001 |
| SAQ–Summary score | 75.15 (0.64), [73.89–76.42] | 69.19 (0.31), [68.58–69.80] | 5.96 [4.55 to 7.38] | <.001 |

**Note:** Values are adjusted means from ANCOVA controlling for baseline scores. Mean differences were calculated as intervention minus control. HC3 heteroscedasticity-robust standard errors were used. Group-by-baseline interactions were included for MMAS-8 and SAQ–Angina frequency. CI indicates confidence interval; BMQ, Beliefs About Medicines Questionnaire; SEAMS, Self-Efficacy for Appropriate Medication Use Scale; SAQ, Seattle Angina Questionnaire.

**Table A 6.2. Difference-in-Differences Sensitivity Analysis of Changes From Baseline to 12 Weeks**

| Outcome | Intervention group (n=46) | Control group (n=46) | DID estimate (95% CI) | P value |
| --- | --- | --- | --- | --- |
|  | Mean change (SE), 95% CI | Mean change (SE), 95% CI | Intervention minus control |  |
| Primary outcome | | | | |
| MMAS-8 score | 1.28 (0.12), [1.04–1.52] | 0.20 (0.11), [−0.01–0.42] | 1.08 [0.75 to 1.40] | <.001 |
| Secondary outcomes | | | | |
| Beliefs About Medicines Questionnaire | | | | |
| BMQ–Necessity | 2.52 (0.27), [1.99–3.06] | 0.63 (0.23), [0.17–1.09] | 1.89 [1.19 to 2.60] | <.001 |
| BMQ–Concerns | −4.33 (0.26), [−4.83–−3.82] | −1.20 (0.21), [−1.61–−0.78] | −3.13 [−3.78 to −2.48] | <.001 |
| BMQ–Necessity–Concerns Differential | 6.85 (0.43), [5.99–7.70] | 1.83 (0.33), [1.17–2.48] | 5.02 [3.94 to 6.10] | <.001 |
| Self-Efficacy for Appropriate Medication Use | | | | |
| SEAMS | 4.78 (0.39), [4.01–5.55] | 0.74 (0.19), [0.36–1.12] | 4.04 [3.19 to 4.90] | <.001 |
| Seattle Angina Questionnaire | | | | |
| SAQ–Physical limitation | 12.42 (1.06), [10.32–14.51] | 4.49 (0.57), [3.37–5.62] | 7.92 [5.54 to 10.30] | <.001 |
| SAQ–Angina stability | 14.67 (2.56), [9.60–19.75] | 2.17 (3.22), [−4.23–8.58] | 12.50 [4.33 to 20.67] | .003 |
| SAQ–Angina frequency | 15.22 (1.03), [13.17–17.26] | 9.57 (1.09), [7.41–11.72] | 5.65 [2.68 to 8.63] | <.001 |
| SAQ–Treatment satisfaction | 14.07 (0.88), [12.33–15.81] | 14.19 (1.01), [12.20–16.19] | −0.13 [−2.78 to 2.52] | .924 |
| SAQ–Quality of life | 26.81 (1.48), [23.87–29.75] | 17.39 (1.43), [14.55–20.23] | 9.42 [5.33 to 13.51] | <.001 |
| SAQ–Summary score | 13.53 (0.71), [12.13–14.93] | 7.72 (0.41), [6.90–8.54] | 5.81 [4.19 to 7.44] | <.001 |

**Note:** Mean change was calculated as the 12-week score minus the baseline score. The DID estimate was the mean change in the intervention group minus the mean change in the control group. DID estimates were obtained by regressing individual change scores on group and using HC3 heteroscedasticity-robust standard errors. All P values are 2-sided. Because only one preintervention measurement was available, the parallel-trends assumption could not be empirically assessed; this analysis should therefore be interpreted as a supportive change-score sensitivity analysis rather than a definitive causal DID analysis. CI indicates confidence interval; DID, difference in differences; BMQ, Beliefs About Medicines Questionnaire; SEAMS, Self-Efficacy for Appropriate Medication Use Scale; SAQ, Seattle Angina Questionnaire.
